# Playback of broadband vocalizations of female mice suppresses male ultrasonic calls

**DOI:** 10.1371/journal.pone.0273742

**Published:** 2023-01-05

**Authors:** Kayleigh E. Hood, Eden Long, Eric Navarro, Laura M. Hurley

**Affiliations:** 1 Department of Biology, Indiana University, Bloomington, Indiana, United States of America; 2 Center for the Integrative Study of Animal Behavior, Indiana University, Bloomington, Indiana, United States of America; Claremont Colleges, UNITED STATES

## Abstract

Although male vocalizations during opposite- sex interaction have been heavily studied as sexually selected signals, the understanding of the roles of female vocal signals produced in this context is more limited. During intersexual interactions between mice, males produce a majority of ultrasonic vocalizations (USVs), while females produce a majority of human-audible squeaks, also called broadband vocalizations (BBVs). BBVs may be produced in conjunction with defensive aggression, making it difficult to assess whether males respond to BBVs themselves. To assess the direct effect of BBVs on male behavior, we used a split-cage paradigm in which high rates of male USVs were elicited by female presence on the other side of a barrier, but which precluded extensive male-female contact and the spontaneous production of BBVs. In this paradigm, playback of female BBVs decreased USV production, which recovered after the playback period. Trials in which female vocalizations were prevented by the use of female bedding alone or of anesthetized females as stimuli also showed a decrease in response to BBV playback. No non-vocal behaviors declined during playback, although digging behavior increased. Similar to BBVs, WNs also robustly suppressed USV production, albeit to a significantly larger extent. USVs suppression had two distinct temporal components. When grouped in 5-second bins, USVs interleaved with bursts of stimulus BBVs. USV suppression also adapted to BBV playback on the order of minutes. Adaptation occurred more rapidly in males that were housed individually as opposed to socially for a week prior to testing, suggesting that the adaptation trajectory is sensitive to social experience. These findings suggest the possibility that vocal interaction between male and female mice, with males suppressing USVs in response to BBVs, may influence the dynamics of communicative behavior.

## Introduction

Vocal communication during sexual interaction has primarily been studied from the perspective of males sending signals to female receivers. However, females also produce signals during intersexual interactions [1–8]. Males that perceive female signals and modify their reproductive behavior accordingly are more likely to successfully reproduce than males that do not respond to female signals [2, 6, 7, 9]. For example, female satin bowerbirds (*Ptilonorhynchus violaceus*) prefer males with intense courtship displays but can be startled by those displays. Males that produce intense displays but reduce display intensity when a female is startled have greater reproductive success than males that do not respond to startle [9, 10]. Similarly, female pholcid spiders (*Physocylus globosus*) stridulate during courtship squeezes by males; males that decrease the number of courtship squeezes after female stridulation have increased fertilization success [6].

In rodents, female behaviors may also influence the outcomes of reproductive interactions. When female golden hamsters (*Mesocricetus auratus*) are unable to perform a pelvic adjustment response of the lordosis posture during copulation, males ejaculate at 1/10^th^ the rate of males with females able to perform the pelvic adjustment response [11]. Male rats are more likely to successfully impregnate a female if the female is able to pace the sexual interaction by escaping to a portion of the arena inaccessible to the male [12]. When female mice (*Mus musculus*) are allowed to escape males during sexual interactions, they accept male approaches more often, indicating that female mice may also be pacing sexual interactions [13]. However, there are no differences in the frequency of intromission depending on whether or not females can escape, suggesting female mice may be able to control the timing of sexual behavior in both conditions [13]. This is consistent with observations of mouse sexual behaviors in a semi-naturalistic enclosure, where females display darting behavior after being mounted, controlling the timing of mounts even in an environment that does not allow them to be physically separate from males [14]. These studies provide important evidence that male rodents may gain reproductive benefits by responding to female signals.

Vocal signals are an additional important part of the repertoire of the behaviors of female mice during opposite-sex interactions. Although males produce a majority of ultrasonic vocalizations in such interactions, females produce a significant minority of these signals [15] that correspond to behaviors by males and females [16]. An entirely different category of signals consists of human-audible and harmonically structured calls known as squeaks, low -frequency harmonic calls, or broadband vocalizations (BBVs) [17, 18]. BBVs are produced by males and females across a variety of contexts including during distress and in social interactions [17, 18], but in opposite-sex interactions are produced predominantly by females [19]. The BBVs produced in the early stages of intersexual interactions are closely paired in time and number with female rejection behaviors such as kicking and lunging, and correspond inversely to male USVs [17, 20]. The number of these early BBVs also negatively predicts later mating success, since males experiencing high levels of BBVs and other female rejection behaviors are less likely to later mount females than males that do not experience high levels of these behaviors [17]. BBVs may therefore signal that a female is not receptive at a given point in time. However, in direct interactions between males and females, it is not possible to determine whether BBVs themselves have any effects on male courtship behavior, because of co-occurring female rejection behaviors such as kicking.

In order to assess the direct influence of BBVs on male mouse sexual behavior, we designed a novel behavioral paradigm to measure the impact of BBV playback on male sexual behavior in the absence of other rejection cues. We predicted that males would respond to BBV playback by decreasing the frequency and intensity of their female-directed behaviors, including USVs. Our results support our prediction in that males decreased the rate of USV production, although they continued to vocalize at a lower rate. Non-vocal behaviors displayed by males were not suppressed in response to BBV playback, suggesting that female BBVs are effective in reducing male vocal courtship.

## Materials and methods

### Animals

This study was carried out in accordance with the recommendations in the Guide for the Care and Use of Laboratory animals of the National Institutes of Health. The experimental protocol was approved by the Bloomington Institutional Animal Care and Use Committee (protocol 18–025). A total of 70 male and 44 female CBA/J mice (Jackson Labs, Bar Harbor, ME) were used in behavioral trials. At the time of recording males and females were between 8 and 9 weeks of age. Prior to experiments, both males and females were placed in multiple pairwise interactions with 3 or more different individuals of the opposite sex for 10 minutes until all individuals had either mounted (males) or were mounted (females). All mice were given food and water *ad libitum* and kept on a 14:10 light:dark cycle. Experiments and sexual experience took place between 10 AM and 5PM. All females and most males were pair-housed, but 7 males were individually housed for a period of a week prior to behavioral trials to investigate the role of prior social experience on response to playback. In order to identify dominance status in pair-housed males, pairs were tested using a tube dominance test in which males were each placed in opposite ends of a plastic tube with an inner diameter too narrow for the mice to pass over one another. Males were simultaneously released into the tube and the first male to exit the tube was labeled as subordinate while the male remaining in the tube was labeled as dominant, consistent with previous uses of tube-dominance tests [21, 22]. Dominance status was only assigned if one male exited the tube within a minute of the test. Pair-housed males were tested twice on the day of recording and were only categorized as Subordinate or Dominant if they performed as such across both tests.

### Playback experiment design

To assess the effects of BBV playback, males and females novel to the males were placed in a standard mouse cage (12X6X6 inches) with a clear plexiglass barrier dividing the cage in half. The barrier had a small hole (1.3 cm in diameter) at the bottom (3.6 cm from the cage floor) to allow for olfactory and tactile investigation by the mice. Clean bedding was placed on the female side of the cage while soiled bedding from the female’s cage was placed on the male side of the cage, in order to stimulate high rates of USV production. The recording cage was placed into a sound attenuating chamber (IAC Acoustics, Naperville, IL). Both the recording cage and sound attenuating chamber were illuminated with a single light source recessed in the top of the chamber. Males were placed into the cage for 60 seconds or until they began producing USVs before the female was placed on the opposite side of the barrier. No BBVs were produced by either the focal male or female during any recording session. After each recording session, mice were returned to their home cages, the bedding was disposed of, and the cage and barrier were cleaned with soap and water, and then wiped with 70% EtOH.

### Recording

Audio and video recordings were taken during each recording session in order to compare male vocal and non-vocal, as well as female non-vocal, behavior across ‘playback’ and ‘no playback’ conditions for all experiments. An ultrasonic microphone (CM16/CMPA, Avisoft Bioacoustics, Glienicke/Nordbahn, Germany) was placed directly above the arena. This was used in conjunction with an UltraSoundGate 116Hb sound card (250-kHz sample rate). Video was recorded with a Canon VIXIA video camera placed above the test cage, with SuperDVR software (Q-See, Digital Peripheral Solutions Inc., Anaheim, CA) and a Q-see four channel DVR PCI video capture card.

### Playbacks

**Fig 1:**
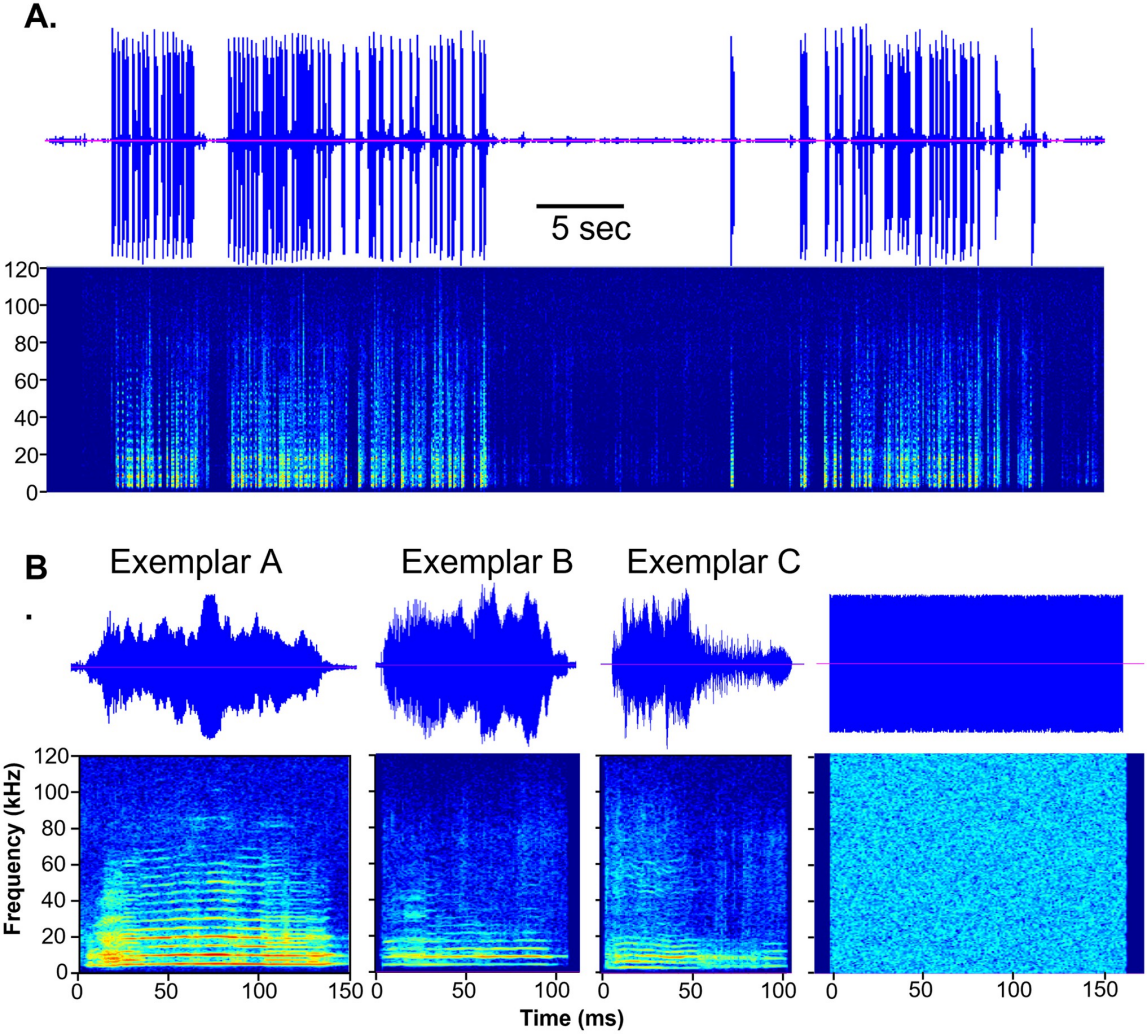
Structure of the stimuli used in the study. **A)** Oscillogram (top) and spectrogram (bottom) of a sequence of exemplar calls over a period of one minute. Each large vertical deviation in voltage in the oscillogram represents a single exemplar call that can be seen in the corresponding spectrogram. Stimuli were all played in the same sequence replicating the timing of a one-minute segment of a direct male-female interaction in which a female was strongly rejecting a male. The one-minute segment was replicated five times to create a five-minute playback. **B)** Oscillograms (top) and spectrograms (bottom) of three exemplar BBVs and WN used as playback stimuli.

In experiments with 36 males, female BBVs were played using an Ultrasonic Dynamic Speaker (Vifa, range from 1–120 kHz), powered by an UltraSoundGate Player 116 (Avisoft Bioacoustics, Glienicke/ Nordbahn, Germany). The speaker was placed directly behind the recording cage and centered so the speaker was equally directed at the female and male sides of the divider. BBVs playbacks were created using a one-minute clip from an ultrasonic microphone recording of direct male-female interaction with a high rate of BBV production and physical rejection of the male by a female [18] (Fig 1A). This minute was low-pass filtered at 40 kHz to remove ultrasound, and to remove ambient noise, the portions of the recording between BBVs were replaced with an equal-length period of silence. The minute-long recording was then repeated four more times to result in a 5-minute high-rate BBV playback that had a natural timecourse of BBV production. Each individual BBV was replaced with one exemplar BBV. The initial exemplar BBV (Fig 1B; Exemplar A) was within one standard deviation of the mean for duration, peak frequency, and percent of non-linear segments for all BBVs in the interaction. Exemplar A was presented to 12 male mice housed in pairs and seven mice housed individually for a week prior to the split-cage assay. To assess whether BBVs with different spectrotemporal structure and amplitude envelopes had different effects on male behavior, two additional calls with varying structure and duration were used (Fig 1B; Exemplars B and C, n = 8 each). Exemplars B and C were used in playbacks to 8 males each housed in pairs. Each of these groups consisted of different individuals. Each individual mouse was presented with only one of the three Exemplar BBV playbacks.

#### Other playbacks

To determine whether the effect of BBV playback on male behavior was specific to the acoustic structure of the BBVs, a group of males were presented with a white noise playback (n = 8; Fig 1B) or no playback (n = 6) in the place of a BBV playback. White noise playback was created by replacing each BBV with a white noise burst of equal duration, with white noise generated with Avisoft SASLab Pro (Avisoft Bioacoustics, Glienicke/ Nordbahn, Germany). ‘No playback’ controls were performed using the same set up as playback trials but with silence in the place of BBVs in the playback file.

For separate set of 9 males, BBVs and USVs were used as playbacks in separate trials in a repeated measures design. The USVs consisted of the same file used in Ronald et al. 2020 [23]. Briefly, USVs were collected from six females that were isolated in an arena and presented with the olfactory cue of male urine. Like the BBV playbacks, USV playbacks were designed to reflect our observations of naturally produced signals in terms of structure, timing, and loudness. For the playback file, a group of USVs with strong signal to noise ratios (eg, S1 Fig) were combined pseudorandomly into clusters of 6, separated by ~100 ms. One cluster was played during minute 1 of the playback, two during minute 2, four during minute 3, two during minute 4, and one during minute 5, for a total of 60 USVs. This pattern was reflective of the USVs produced by females recorded in the study, and playback of this file increased the production of USVs by solo males, when combined with the cue of female urine [23]. Both male and female mice produce USVs during sexual interactions [15]. In order to control for possible contributions of female USVs to the total USV numbers, a group of males (n = 6) experienced the BBV playback procedure described above but with an anesthetized female instead of a live female. An additional set of males (n = 6) were presented with anesthetized females or female bedding without BBV playback for comparison. Females were anesthetized with an intraperitoneal injection of ketamine (120 mg/kg), xylazine (5 mg/kg), and acepromazine (2 mg/kg) at least 20 minutes prior to experimentation. Ophthalmic ointment was applied to prevent the eyes from drying. Females were unresponsive during playback experiments and were placed with their nose in the divider opening. After the completion of the playback trials, females were kept on a source of gentle heat and monitored until recovery.

### Loudness calibration

BBVs were initially recorded using a microphone suspended above an arena containing an interacting male and female mouse, but BBVs may be produced at high intensities, and can be readily audible to a human listener from some distance away. Moreover, females are usually in close proximity to a male when producing a BBV, since they are often concurrently kicking at males or making brief lunges towards males [17, 20]. To estimate the intensity of BBVs from the perspective of male mice, we recorded BBVs from mice held close to a condenser microphone (CM16/CMPA; Avisoft Bioacoustics, Berlin, Germany) via an Avisoft-UltrasoundGate 116H Recorder (#41163; Avisoft Bioacoustics, Berlin, Germany) with a sampling rate of 250 kHz. The same microphone was placed at the approximate position of a mouse’s head within the arena was used to match the BBV playbacks of Exemplar A to the same rms intensity as the close-proximity recording. Exemplar calls and WN varied in rms intensity when measured at the microphone. Relative to each other, Exemplar B had the highest root mean squared (rms) intensity, with Exemplar A at 3.4 dB less intense, Exemplar C at 12 dB less intense, and WN at 6.7 dB less intense. To assess the absolute intensity of played back BBVs, BBV playback was compared to a standard sound source using an ACO Pacific PS9200 sound calibration kit (ACO Pacific, Belmont, CA). Using this approach, Exemplar A reached 104.6 dB at its maximum peak-to-peak value, Exemplar B and Exemplar C reached 100.8 and 98.6 dB, respectively, and WN reached 104.9 dB. USV playback intensity was calibrated by matching the rms intensity of the loudest played USV to the intensity of the same recorded USV, using the same microphone (CM16/CMPA; Avisoft Bioacoustics, Berlin, Germany) and measurement software (Avisoft SASLab Pro, Avisoft Bioacoustics, Berlin, Germany). This resulted in a peak-to-peak intensity of 89.3 dB at 2 cm from the speaker, in comparison with a calibrated sound source (PS9200 sound calibration kit, ACO Pacific, Belmont CA).

### Behavioral analysis

#### Non-vocal behavior

Non-vocal behavior was quantified using ODLog (Macropod Software) by four trained observers who maintained less than 5% inter-observer variation across recordings. Observers measured the duration of time the male spent digging, and grooming, and the duration of time both male and female spent investigating the divider opening in the plexiglass barrier. Digging was noted when males moved bedding with their forelegs. Grooming was noted when males ran their paws or tongue through their fur. Investigation was noted for either the male or female when the mouse put their nose in or within one head length of the opening in the plexiglass barrier. All videos were analyzed without audio, blinding the observer to the treatment of a given recording.

#### Vocal behavior

Vocal behaviors were analyzed in Avisoft SASLab Pro (Avisoft Bioacoustics, Glienicke/ Nordbahn, Germany). Files were high-pass filtered at 40 kHz and trained observers used the whistle tracking function to count USVs. USV counts using Avisoft’s whistle tracking function were accurate within a 5-percent difference threshold of non-whistle tracking counts. Observers also quantified the number of 50 kHz harmonic USVs, which have been correlated with mounting behavior, using the criteria described in Hanson & Hurley 2012 [24]. For USV playbacks, played USVs were not counted.

### Statistical analysis

Statistical analyses were performed in SAS (Version 9.3) and Statistica software (Tibco Data Science, Pao Alto, CA). In order to investigate changes in vocal and non-vocal behaviors in response to playback and other contextual factors such as stimulus type and dominance status, we used general linear mixed models (Proc GLMM) in SAS. Due to the high level of individual variation in USV production, models were run with Male Identity as a repeated measure to identify consistent patterns of changes in behavior across experiments among males with different baselines. Vocal behavior counts and non-vocal behavior durations were summed into 5-minute time periods that correspond to the baseline, playback, and recovery sections of the experiments. Each behavior was modeled as the dependent variable with time period (baseline, playback, recovery), male dominance status, Exemplar BBV type (A, B, or C), and the interaction between time period and Exemplar BBV type included as independent factors. A total of 6 models were run, 2 to investigate changes in vocal behaviors (i.e., USV production, 50 kHz USV production) and 4 to investigate changes in non-vocal behaviors across all BBV Playback subjects. Significant main effects of independent factors were further explored with post-hoc t-tests. Benjamini-Hochberg corrections with a Q value of 0.05 were used to adjust for multiple comparisons [25]. A separate general linear model was used to assess the effect of BBV versus non-BBV stimuli on USV production, using time period as a within-subjects factor and stimulus type (BBV, silence, or WN) as a between-subjects factor. Repeated measures ANOVAs were used to assess changes in USV rates across subsequent one-minute rounds of the playback. To assess the distribution of USVs across time periods in the presence of awake versus anesthetized females, USVs in each time bin were normalized to the total number of USVs in a given trial, and differences assessed with a factorial ANOVA with time bin and condition (anesthetized versus awake female) as cofactors. Fisher’s Least Significant Difference (LSD) test were used for posthoc comparisons of groups in the general linear models.

A Pearson’s correlation was used to establish the relationship between initial USV rate and change in calling rate across trials with BBV playback, and with BBV and WN playback. Residuals from the regression were used to assess the difference between BBV and WN playback, using a one-way ANOVA. Levene’s test for homogeneity of variance was used to assess whether males with high versus low initial USV rates had greater variability in response to BBV playback. An Analysis of Covariance (ANCOVA) was used to compare the regression of male versus female investigation time across the baseline, playback, and recovery time periods. Variation in values is expressed as the standard error of the mean (s.e.m.).

An ***event-triggered average*** of the probability of USVs relative to BBVs was generated for the 3 seconds before and after each BBV for the 12 males presented with Exemplar A. This was accomplished by adapting a custom Matlab (MathWorks) script for event-triggered averaging around communication signals [26]. After the generation of a matrix of the time of each USV relative to each BBV, time differences within ± 3 sec of each BBV were convolved with a Gaussian filter with standard deviation of 0.1 sec. The resulting filtered time differences were summed across all BBVs, and normalized by the number of BBVs to generate a temporal probability distribution of USVs relative to BBVs. To test the null hypothesis that USVs were distributed randomly around the occurrence of BBVs, 95% confidence intervals were generated by randomly shuffling the times of all USVs in the ±3-second windows around BBVs, followed by Gaussian filtering, summation, and normalization. This shuffling process was reiterated 1,000 times, and the 2.5^th^ and 97.5^th^ percentile in each time bin were used as the upper and lower 95% confidence intervals for the null probability distribution.

## Results

**Fig 2:**
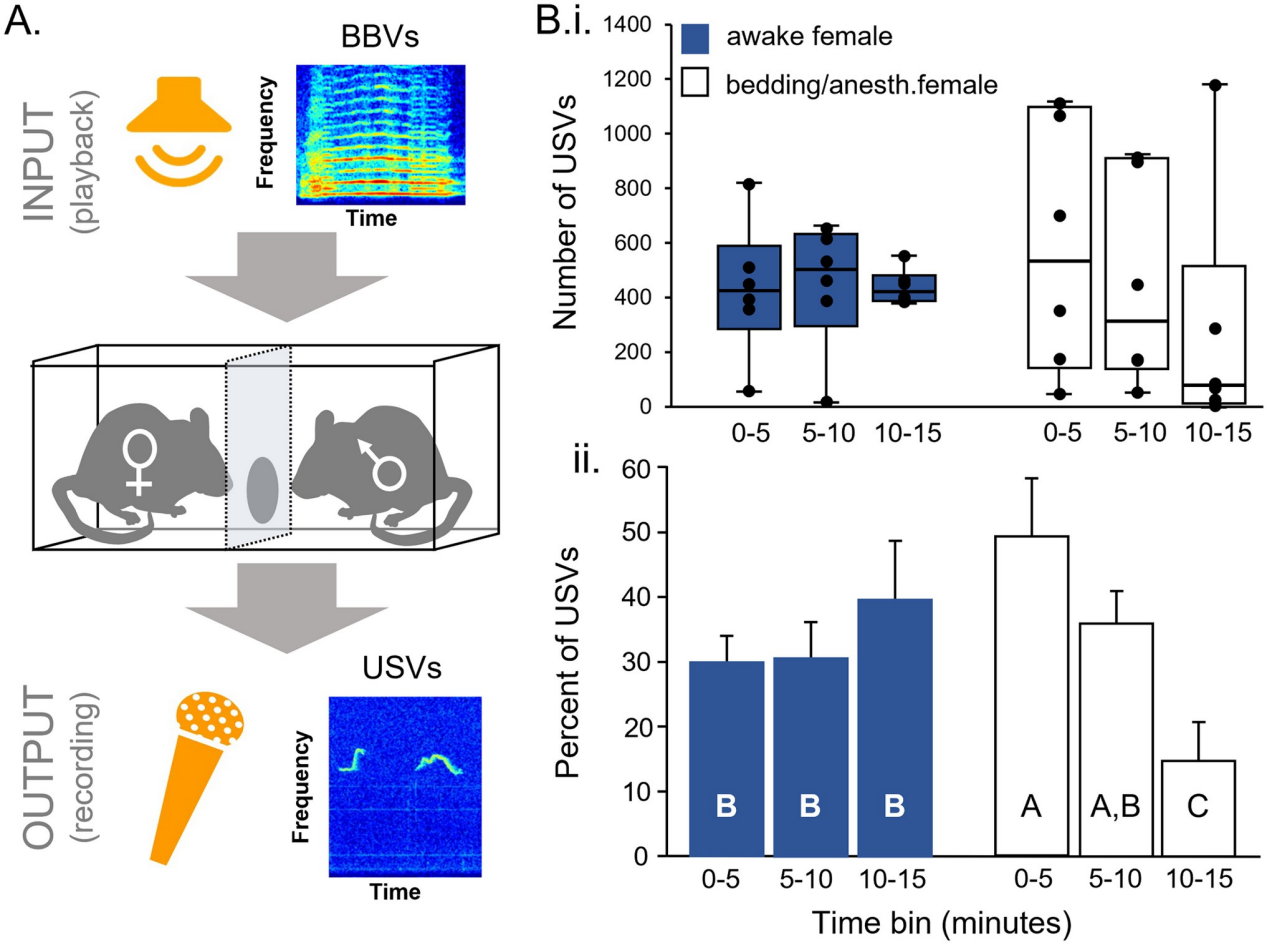
Behavioral assay. **A)** BBVs are played to a male-female pair separated by a barrier, and USVs and non-vocal behaviors are measured as dependent variables. **B)** USV production over 15 minutes in the absence of BBV playback, for males placed in a divided cage with awake females (blue: n = 6) or soiled female bedding or anesthetized females (white: n = 6). **i)** Box and whiskers plots of numbers of USVs within specific time bins. **ii)** Percentage of USVs within specific time bins, normalized to total USV numbers for individual mice; in each treatment, all three time periods sum to 100% for each interaction. When males are paired with awake females, calls are maintained over time. When males are presented with soiled bedding only, or paired with anesthetized females, calls decline over time.

To create a behavioral paradigm in which males produced consistent levels of USVs but that precluded direct interaction with spontaneous BBVs, females were separated from males using a Plexiglas barrier with a small hole for olfactory and tactile investigation (Fig 2A). In this situation, males produced consistent numbers of USVs over a 15-minute period, with roughly equal average percentages of calls in each of three five-minute bins (n = 6, Fig 2Bi, filled bars). In contrast, males paired with anesthetized females on the other side of the barrier or with female bedding alone showed a steady decline in USVs over the 15-minute period, with the highest number of calls in the first time bin (Fig 2Bi, open bars). To assess whether these two distributions were significantly different, the numbers of calls in each time bin was normalized to the total number of USV produced by that male in all three time bins, so that the percentages across all three bins added up to 100% for each male. These distributions for the awake and anesthetized females were significantly different, with an interaction between condition (awake vs anesthetized) and time bin (Fig 2Bii; factorial ANOVA, F_(2,30)_ = 5.79, p = 0.007) Awake females were therefore used as stimulus animals during playback recordings, to avoid the confounding effects of a spontaneous decline in USVs.

### Vocal response to playback

Five-minute sequences of exemplar BBVs were played back to male mice in the split-cage paradigm. Playbacks were preceded and followed by five minutes without playback that served as baseline and recovery periods, creating three five-minute periods: the ***baseline*** from 0 to 5 minutes, the ***playback*** from 5 to 10 minutes, and the ***recovery*** from 10 to 15 minutes. During the playback, one of three exemplar calls (A, B, and C) were used to replace naturally occurring calls in a one-minute bout taken from a direct interaction between a male and female that exhibited a high level of female rejection behaviors [17] (Fig 1A). The numbers of USVs produced among experiments with individual males varied widely, from 157 to 3214 USVs produced in the 15-minute time period, averaging 1128.5 ± 149.8 USVs. A specific type of call associated with mounting behavior in direct opposite-sex interactions, 50-kHz harmonic USVs [24], occurred at a lower rate, averaging 58.1 ± 13.4 calls per trial.

**Fig 3:**
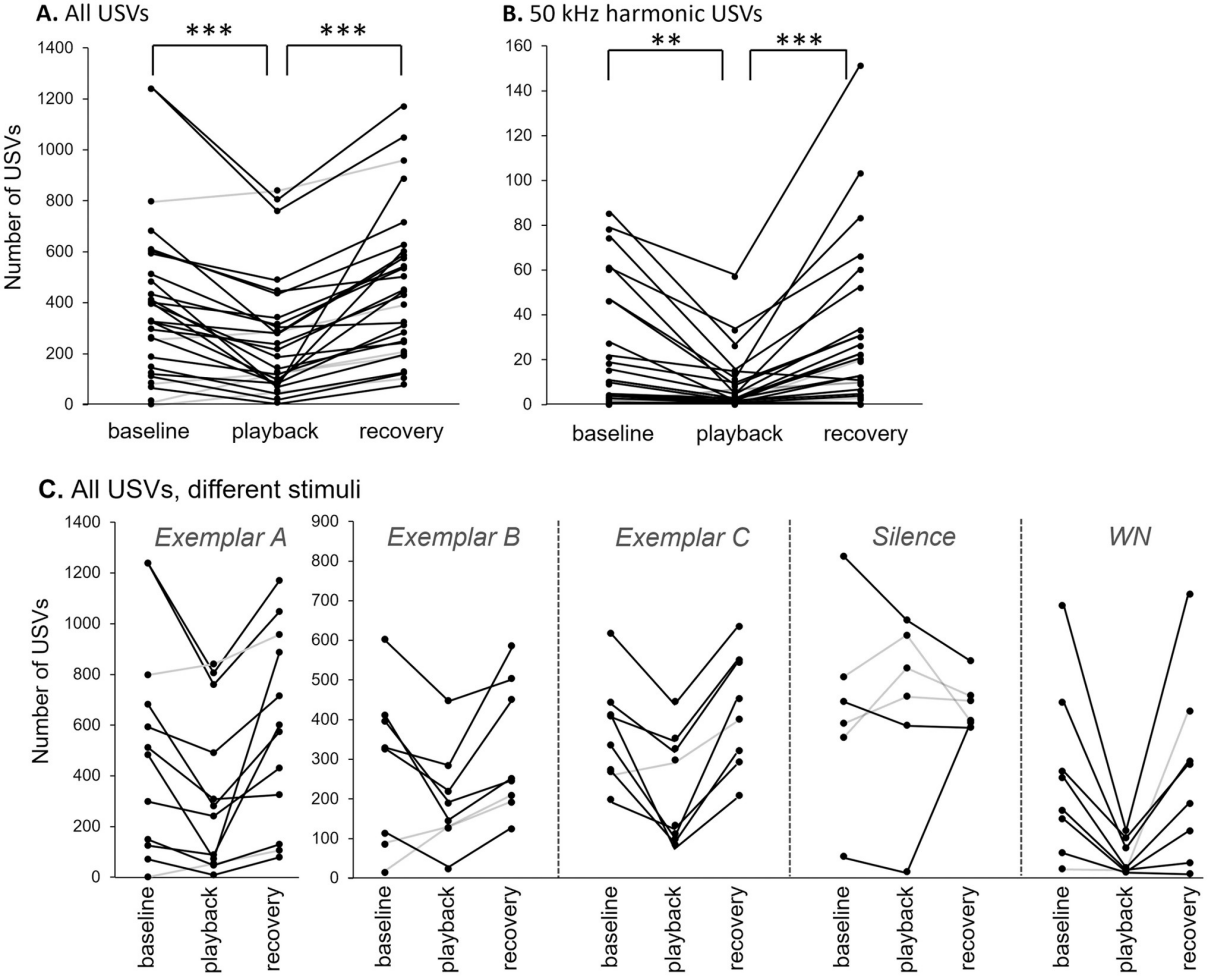
Effect of BBV playback on the number of USVs produced for A) all types of USVs and B) ‘50-kHz harmonic’ USVs in response to the three exemplars (A-C). BBV playback significantly decreased USV numbers during the playback, and USV numbers recovered significantly following playback. Box-and-whiskers plots show the median, and quartile values for the baseline period (0–5 minutes), the playback period (5–10 minutes), and the recovery period (10–15 minutes). Data points represent values for individual males (n = 28). C) Plots showing total USV numbers for responses to specific stimulus types, including all exemplars (Exemplar A: n = 12; Exemplars B and C: n = 8 each), silence (n = 6), and WN (n = 8). For all plots, gray lines indicate individual mice that increased calls during the playback relative to baseline.

There was a significant main effect of time period for both vocal behaviors (Table 1), USV production (F_(2,85)_ = 23.48, p<0.0001) and 50 kHz Harmonic USV production (F_(2,74.7)_ = 15.05, p<0.0001). Post-hoc comparisons identified a significant decrease in USV production during the playback time period when compared to baseline (t_84.5_ = 4.21, p<0.0001) and recovery (t_84.5_ = -5.87, p<0.0001) periods (Fig 3A). However, there were no significant effects of exemplar type (A, B, or C; F_(2,28.1)_ = 1.56, p = 0.2281) or the dominance status of the males (dominant versus subordinate; F_(1, 25.9)_ = 0.39, p = 0.5359) on total USVs. 50-kHz harmonic calls occurred at a low rate relative to total USVs, making up 4.7 ± 1.1% of calls in the baseline period. When 50-kHz harmonic calls were considered as a separate category, the effect of playback was similar to its effect on USVs as a whole. Of 14 trials in which 10 or more 50-kHz harmonics were produced in the baseline period, 50-kHz harmonic USVs decreased to 34.7 ± 6.4 percent of their baseline values during playback and recovered to 142.5 ± 25.4 percent of baseline values. Production of 50 kHz harmonic USVs also significantly decreased in the playback period compared to the baseline (t_72.8_ = 3.09, p = 0.0028) and recovery (t_72.8_ = -5.06, p <0.0001) periods. Like USVs as a whole, exemplar type and dominance status had no effect (Fig 3B; Table 1).

**Table 1:** Output of a general linear mixed model assessing the effects of BBV playback.

	all USVs		50 k harm		dig		groom		divider opening (m)		divider opening (f)		
Effect	*df*	*F*	*p*	*F*	*p*	*F*	*p*	*F*	*p*	*F*	*p*	*F*	*p*
**Time Bin**	2	23.48	***<0*.*0001***	15.05	***<0*.*0001***	7.51	***0*.*001***	1.34	0.2692	4.16	0.0194	1.11	0.3356
**Exemplar Type**	2	1.56	0.2281	0.85	0,4385	0.52	0.6023	0.25	0.7827	3.18	0.0584	0.1	0.9015
**Social Status**	1	0.39	0.5359	0.02	0.899	0.26	0.6153	1.01	0.3263	3.67	0.0679	0.99	0.3266
**Exemplar X Bin**	4	1.08	0.3696	0.78	0.5434	6.23	***2E-04***	1.82	0.1256	6.57	*1E-04*	6.19	***2E-04***

In addition to BBV exemplars, there were two additional stimulus conditions- the presentation of silence, or of WN bursts, in the place of BBVs. Although no decreases in USVs occurred during 5 minutes of silence in the place of playback (Fig 2, filled bars), WN decreased USVs to 14.2 ± 3.9% of their baseline values, with a recovery to 64.3 ± 15.0% of baseline. To assess whether the type of stimulus (BBVs, silence, or WN) influenced USV suppression, we used an additional general linear mixed model with male identity as a repeated factor and time bin (baseline, playback, recovery), stimulus type (BBVs, silence, or WN) and the interaction between time period and stimulus type as independent factors. There was a main effect of time period (F_(2,111)_ = 12.22, p < 0.0001), with the numbers of USVs during playback significantly less than in the baseline period (t_110_ = 3.82, p = 0.0002) or recovery period (t_110_ = -3.73, p = 0.0003). There was no main effect of stimulus type (F_(2,44.2)_ = 2.22, p = 0.1211). However, there was an interaction between stimulus type and time bin (F_(4,111)_ = 3.38, p = 0.012), so that playback periods were different from baseline for BBV playback (p < 0.0001) and for WN playback (p = 0.002), but not for silence (p = 0.712). Thus, the playback of BBVs and WN decreased USVs, but an equivalent period of silence did not. Responses to all tested stimuli including the threeexemplars, WN, and silence, are shown in Fig 3C.

**Fig 4:**
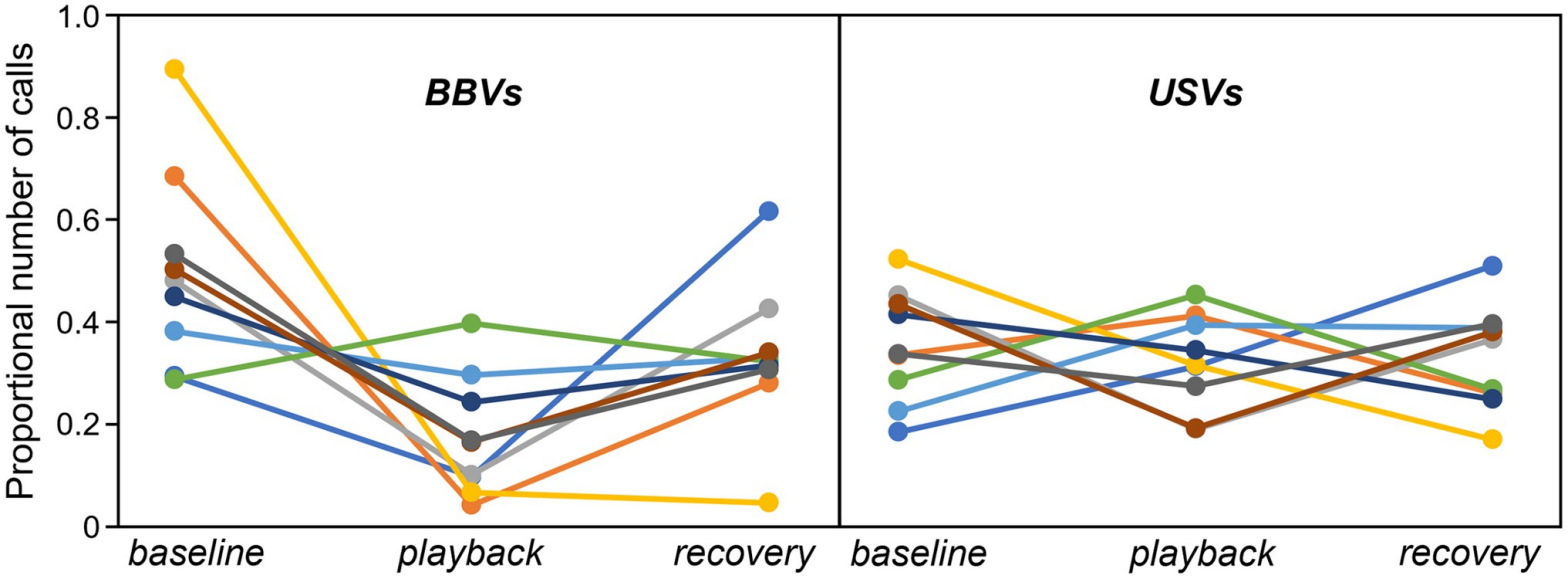
Plot of responses to playback of BBVs (left panel) versus USVs (right panel) in the same individuals (n = 9). Responses are presented as proportional USV numbers relative to all USVs in a given trial; for each trial, proportions in the three time bins (baseline, playback, and recovery) summate to 1. Specific colors represent responses of the same individuals to BBVs and USVs.

A separate group of nine males were exposed to playbacks of both BBVs and a set of USVs collected from female mice [23] in separate trials. A repeated measures ANOVA using both the time bin (baseline, playback, and recovery) and the call type as within-subjects factors did not show a main effect of call type or of bin on the USV number (call type: (F_(1,8)_ = 0.001, p = 0.975; bin: F_(2,7)_ = 1.97, p = 0.21). However, there was a significant interaction between the bin and call type (F_(2,7)_ = 9.173, p = 0.011, such that calls decreased during the BBV playback, but not the USV playback, relative to the baseline condition (Fig 4).

### Variability among males

**Fig 5:**
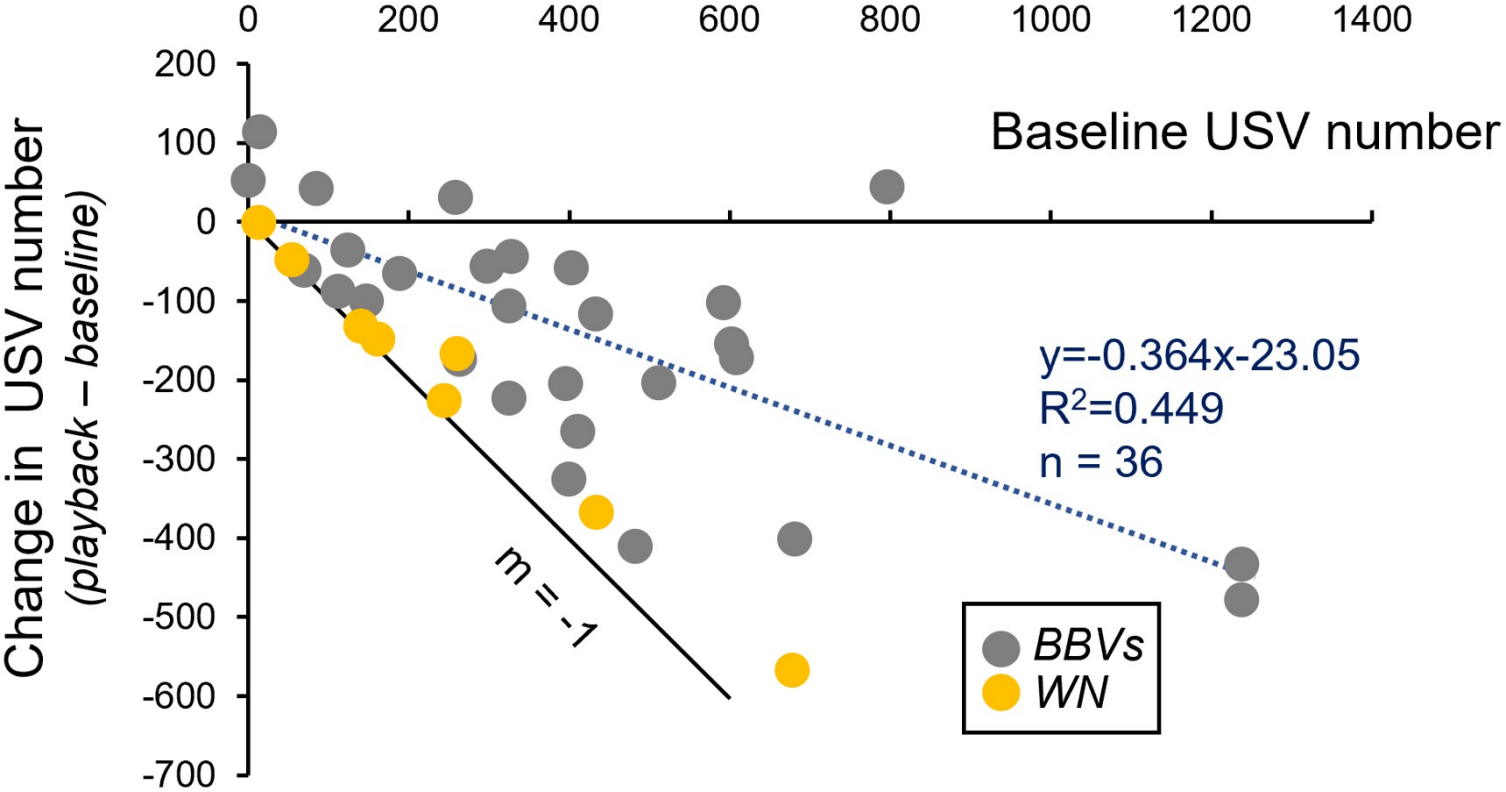
Comparison of changes in USV number induced by BBV playback relative to the baseline call number. Gray symbols mark trials using the 3 exemplar calls, and yellow symbols indicate trials using white noise (WN). The dashed line indicates the linear regression for all data points in the scatterplot. The solid line with a slope of -1 designate the maximum possible effect in which calls are totally suppressed during BBV playback.

There was significant inter-male variability in both the overall numbers of calls produced, and in the responses to BBVs. We therefore assessed whether the variation in calls during the baseline period predicted the responses to playback for the 28 trials with BBV playbacks (Fig 5, gray circles). For example, interactions starting at low USV rates may be constrained from sizable decreases, creating floor or boundary constraints, but could potentially show some changes in the opposite direction, to increase USV number in some males. Fig 5 shows the relationship between USV numbers in the baseline period versus the difference in USV number from the baseline to the playback period. Interactions with very low baseline numbers were constrained from large decreases, but some showed modest increases in call number. At higher baseline values, males showed a range of responses, with some males decreasing USV number almost entirely, indicated by their proximity to a line with a slope of –1 in Fig 5. However, some males decreased their USV rate much less. BBV playback therefore did not cause most males to stop producing USVs, but instead to modulate their rate of USV production, with varying amounts of modulation among males.

Although white noise reduced USVs, similar to the playback of calls, there were quantitative differences in the effects of the two stimulus types. This is depicted in Fig 5, which in addition to plotting responses to BBV playback (gray circles), plots responses to WN (yellow circles). The responses to WN were consistently at the maximum end of the range for playback-induced decreases. To quantify this difference in trajectory between BBV and WN presentation, we calculated the regression for all playback groups (dashed line), and compared the residuals for the trials using BBVs versus WN. Residuals for the WN were significantly more negative than residuals for the exemplar playbacks (one-way ANOVA, F_(1,34)_ = 8.58, p = 0.006), establishing that WN and BBVs evoked qualitatively similar but quantitatively distinct levels of USV suppression.

### Timecourse of vocal response to BBV playback

To assess the interaction between USVs and the BBV playback on a finer time scale, we more closely examined the relationship between BBVs and USVs during the playback period. Playbacks consisted of 5 repetitions of a one-minute playback module, each of which contained two intensive bursts of BBVs (Fig 1A). This allowed us to examine the response to five identical rounds of playback over time. We further divided the playback period into 5-second bins (60 bins total, or 12 bins per module). Because of the density of the call bursts in the playback, this resulted in a bimodal split in the numbers of BBVs across 5-second bins. Each one-minute module of the playback contained 6 bins with 3 or fewer BBVs, and 6 bins with 11 to 15 BBVs. These parameters allowed us to compare the USV response during equal durations of high versus low BBV playback in each one-minute repetition.

For this analysis, we also included seven males that had been housed individually for a week prior to experiments as opposed to with a same-sex social partner. These males were excluded from the general linear model above because there was no opportunity for them to be dominant or subordinate and because they were only exposed to a single call type, Exemplar A. We therefore compared these only with the 12 socially housed mice exposed to Exemplar A. Interactions with males housed individually produced a quantitatively but not significantly higher number of total USVs than socially housed males (individually housed: 2125.3 ± 306.8; socially housed: 1433.1 ± 335.6, one-way ANOVA F_(1,17)_ = 2.1, p = 0.17). Similar to the socially housed males, BBV playback significantly decreased USV number for the individually housed males to 66.5 ± 13.5 per cent of the baseline value, with recovery to 100.4 ± 12.7 of the baseline (repeated measures ANOVA with time bin as a within-subjects variable, F_(2,12)_ = 4.67, p = 0.032).

**Fig 6:**
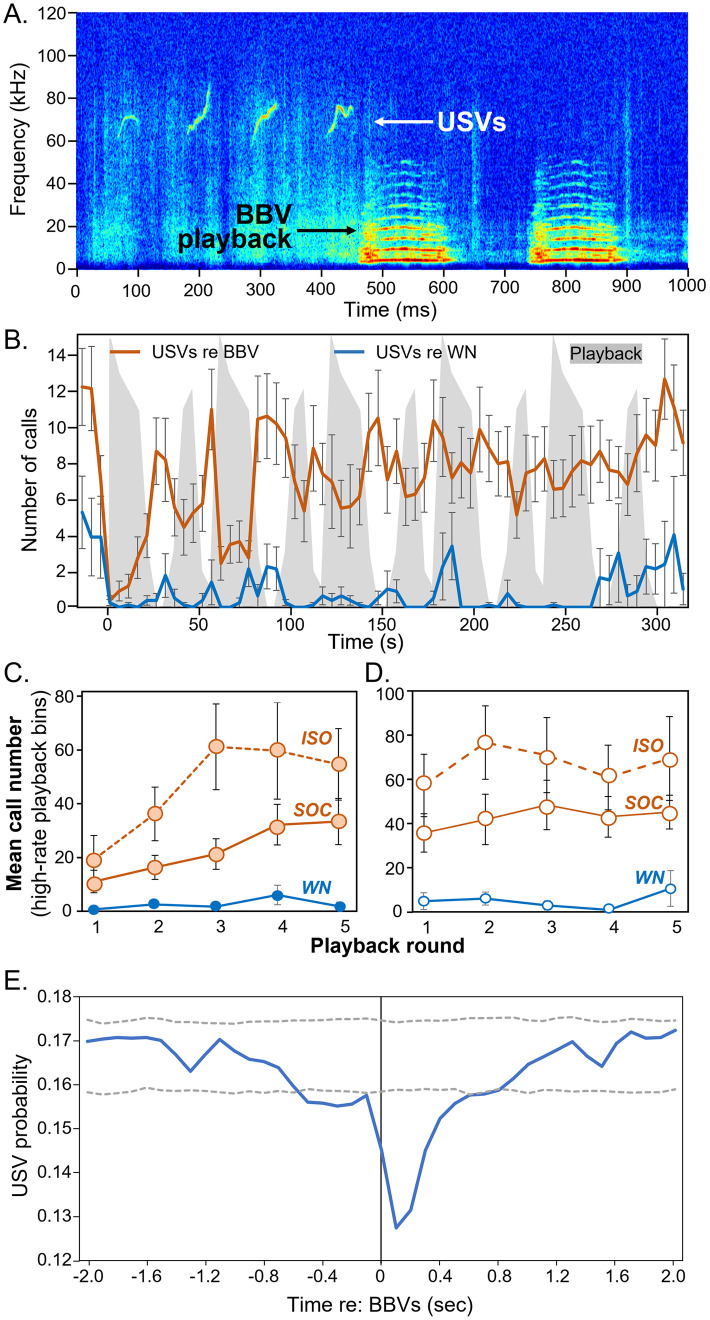
Interaction of BBV playback with USV production during the playback period. **A)** Clip of one experiment showing cessation of USVs at the onset of BBV playback. **B)** Interleaving of BBVs and USVs during the 5 minutes (300 s) of playback, in 5s bins. Three time bins before and after the onset of playback are also represented. Gray shade represents numbers of played stimuli in 5s bins of the playback. Bins contained either 3 or fewer, or 11 or more, BBVs. Orange line shows average USVs in each 5s bin for experiments using Exemplar A (n = 19). Blue line represents USV values in response to WN (n = 8). **C)** Average USV numbers in 5s bins with high BBV numbers in 5 subsequent playback rounds. Mice housed individually (ISO) habituate to BBV playback sooner, while mice exposed to WN do not habituate. **D)** Average USV numbers in 5s bins with low BBV numbers in 5 subsequent playback rounds (white bins in (B). USV numbers in these bins do not habituate over playback rounds. **E)** Event-triggered average of the probability of USVs (blue line) in the two seconds before and after BBVs, for the 12 males presented with Exemplar A. Dashed lines indicate upper and lower confidence intervals.

Fig 6A shows an example of a playback in which USV production coincided with the beginning of BBV playback. Fig 6B plots the numbers of BBVs in 5-second bins over the timecourse of the playback (shaded regions), with double bursts of BBVs visible for each identical one-minute round. Three five-second time periods are also included to represent responses before and after the playback. USVs over this time period (orange line) showed two interesting patterns. First, they were interleaved with the BBV playbacks, so that USV numbers were smaller when BBV numbers are higher (repeated measures ANOVA of total USVs in high-BBV versus low-BBV bins across mice: F_(1, 17)_ = 14.27, p < .001). Second, the effect of BBVs were most pronounced in the earliest rounds of the playback, when USV numbers decline to near an average of zero during playback. In later rounds of the playback, decreases in USVs during BBV bursts were not as extreme. We quantified this adaptation by measuring the numbers of USVs during the high-intensity playback bins (shaded areas in Fig 6B) in each round separately. Fig 6C shows the increase in average USVs in these bins across the 5 playback rounds for mice that were housed socially (solid orange line) versus individually (dashed orange line). A repeated measures ANOVA with ‘round’ as a within-subjects factor and ‘housing condition’ as a between-subjects factor returned a significant effect of ‘round’ (F_(4,68)_ = 13.93, p < .001), and a ‘round X housing condition’ interaction (F_(4,68)_ = 2.91, p = .028), but not of ‘housing condition’ (F_(1,17)_ = 2.76, p = 0.115). For the socially housed mice, numbers were significantly different from Round 1 by Rounds 4 (Fisher’s LSD, p = 0.001) and 5 (p < 0.001). For the individually housed mice, numbers were significantly different from Round 1 by Round 2 (p = 0.039), and Rounds 3–5 (p < 0.001 for each). Thus, individually versus socially housed mice showed a different trajectory of adaptation to BBV playback. In comparison, performing the same analysis for time bins with low BBV numbers (Fig 6D) showed no effects of Round (F(4,68) = 1.54, p = 0.199), housing condition (F_(1,17)_ = 1.817, p = 0.195), or their interaction (F_(4,68)_ = 0.546, p = 0.702). Mice housed in isolation for a week therefore showed more rapid adaptation to the BBV playback than mice housed socially over the same period of time.

We assessed the timing of USVs around BBVs on an even finer time scale by applying an event-triggered averaging process [26] to each individual BBV for the 12 males exposed to Exemplar A, using the start times for BBVs and USVs as event markers. The average probability of USVs declined rapidly following BBVs and remained below the 2.5% confidence interval for over half a second (Fig 6E).

### BBV playback with anesthetized females

**Fig 7:**
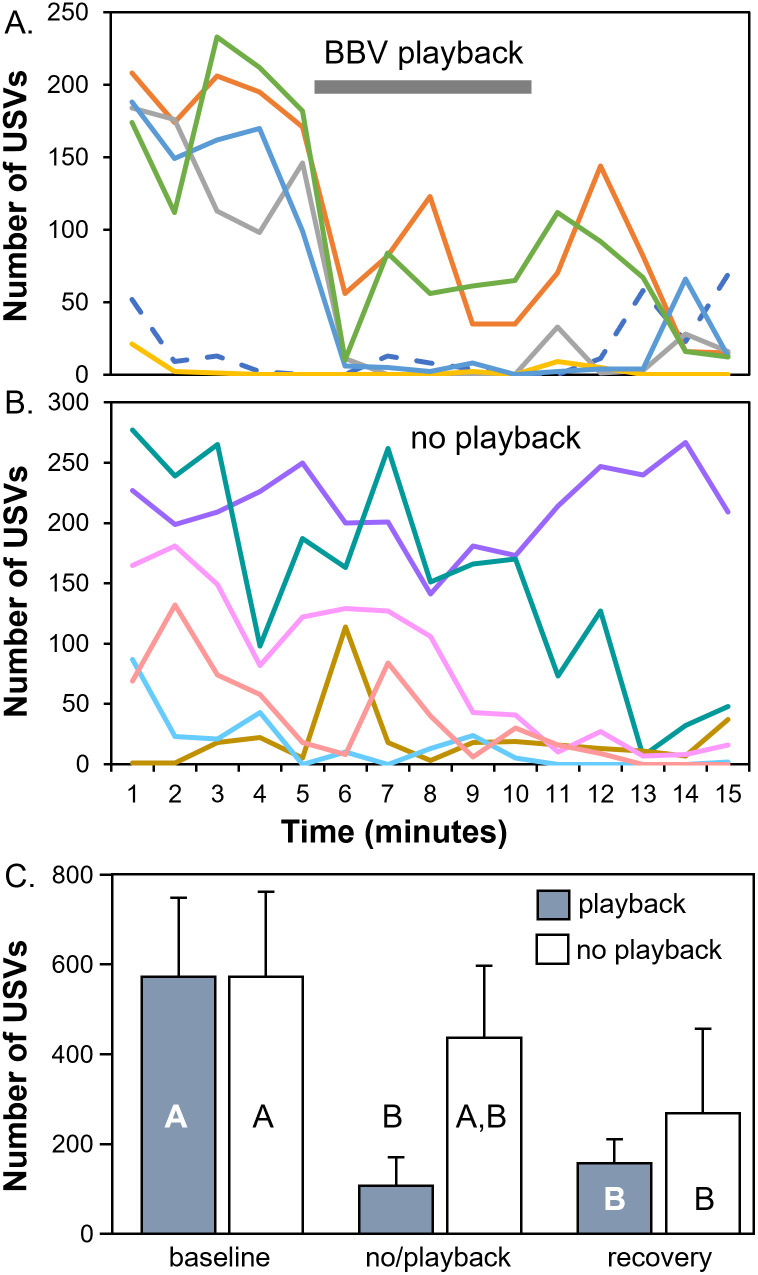
Comparison of numbers of USVs produced with **A)** BBV playback (n = 6) and **B)** no playback (n = 6) in males presented with soiled female bedding only (dashed line), or paired with anesthetized females (solid lines). (A) and (B) show 1-minute bins, with the gray bar in (A) marking the duration of playback. The onset of playback was associated with a sharp decrease in the numbers of USVs in interactions with robust USVs in the first five minutes. Interactions in (B) also appear in Fig 2. Colors represent different individual trials: different males were used for trials in (A) and (B). **C)** Average USV numbers for the three five-minute time bins for the trials represented in A) and B), with gray = playback and white = no playback trials. For playback trials, a significant decrease occured in the playback relative to the baseline time period, something that did not occur for no-playback trials. Letters represent Fisher’s least significant difference posthoc tests for a repeated measures ANOVA, as described in the text. Columns with different letters are significantly different from each other.

Since female mice produce a minority of USVs during opposite-sex interactions, we assessed whether BBVs induced decreases in USVs when females were unable to produce USVs because they were anesthetized, or in one case because no female was present. We therefore compared trials in which females were anesthetized and no playback was presented (these were the same trials presented in Fig 2B, open bars), with trials in which females were anesthetized, and a playback of Exemplar A was presented. Fig 7A and 7B presents the minute-by-minute USV counts in each individual trial for the playback (Fig 7A; n = 5 for trials with anesthetized females [solid lines], and n = 1 for trial with female bedding only [dashed line]) and for the no-playback (n = 6) conditions. In the playback condition (Fig 7A), mice with high numbers of BBVs in the baseline show a sharp decrease in the minute following the onset of BBV playback, even though little recovery followed the playback. A repeated measures ANOVA with time period as a within-subjects factor and condition (playback vs no playback) as a between-subjects factor showed an effect of time bin (F_(2,20)_ = 11.20, p = 0.0005), but no main effects the condition (F_(1,10)_ = 0.64, p = 0.44) or a bin X condition interaction (F_(2,20)_ = 2.16, p = 0.14). However, average USV numbers in the playback bin were significantly lower than in the no playback bin for the playback condition (Fisher’s least significant difference, p = 0.040), but not the no-playback condition (Fig 7C).

### Non-vocal response to BBV playback

**Fig 8:**
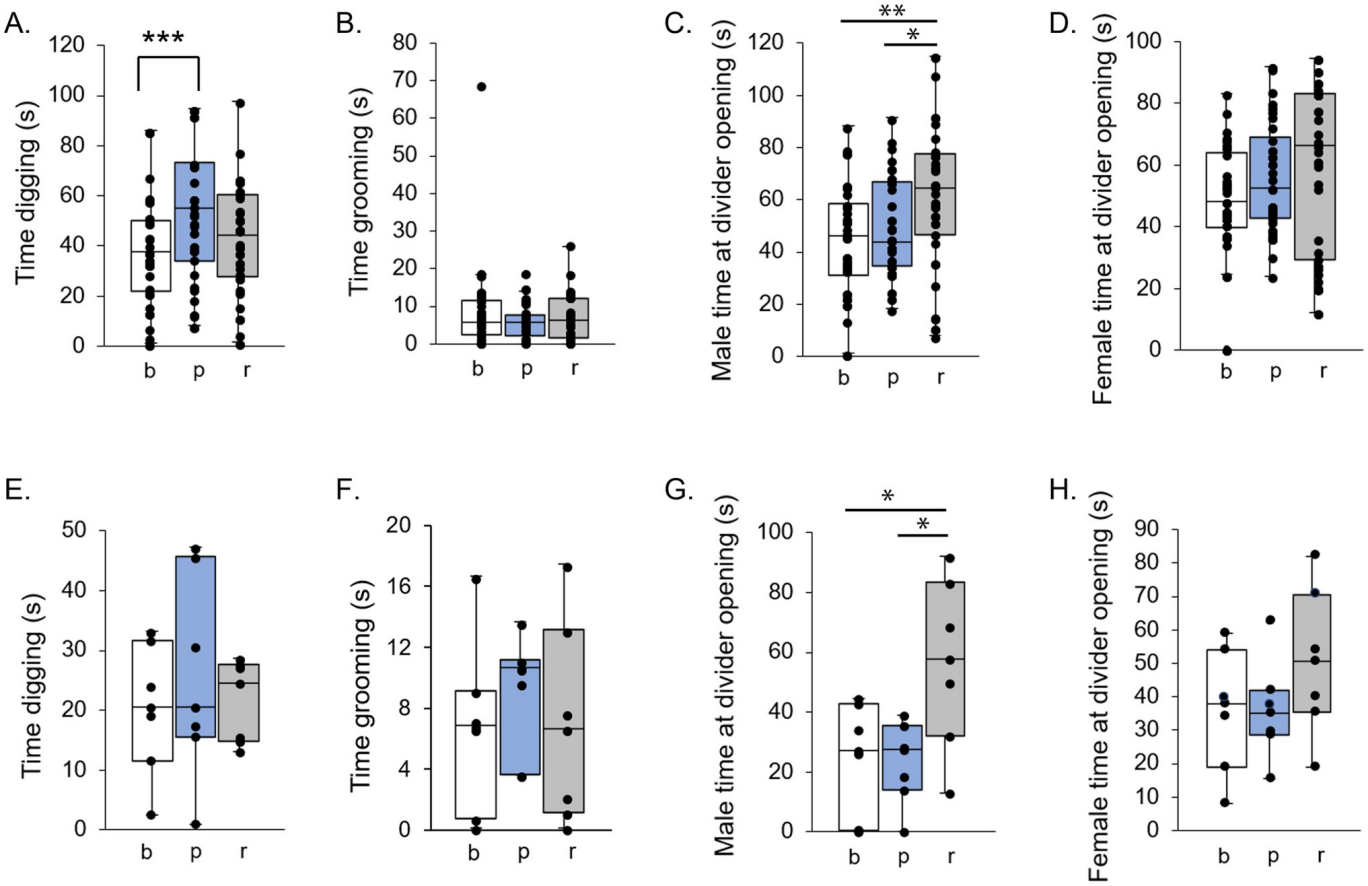
Effect of playback on nonvocal behaviors. Time spent digging **(A. BBV Playback, E. White Noise)**, time spent grooming **(B. BBV Playback, F. White Noise)**, time spent at the divider opening by males **(C. BBV Playback, G. White Noise)** and time spent at the divider opening by females **(D. BBV Playback, H. White Noise)**. A significant main effect of time period (baseline, b, playback, p, or recovery, r) was found for digging for BBV Playback. A significant main effect of condition (BBV Playback or White Noise) was found for digging and male time at the divider opening. Post-hoc comparison p-values are represented as * = p<0.05; ** = p<0.01; *** = p<0.001.

To assess whether BBV playback influenced non-vocal behaviors, the duration of digging and grooming by males, and investigation of the divider opening by both males and females were combined into 5-minute bins (Fig 8A–8D). The only non-vocal behaviors to have a significant main effect of time period on behavior duration were Male at divider opening (F_(2,74.3)_ = 4.16, p = 0.0194) and Digging (F_(2, 78.4)_ = 7.51, p = 0.001). Digging was the only non-vocal behavior to change significantly from baseline during the playback (t_76.7_ = -3.77, p = 0.0003). Male at divider opening significantly increased relative to baseline (t_93.5_ = -2.74, p = 0.0074) and playback (t_72.9_ = -2.44, p = 0.0171) during the recovery time period (Fig 8C). Males showed no significant change in grooming duration across time periods (Fig 8B; F_(2,60.8)_ = 1.34, p = 0.27). Unlike males, females had no difference in the duration of investigation across the baseline, playback, and recovery time periods (Fig 8D; F_(2,75.1)_ = 1.11, p = 0.34). For digging and male and female investigation, there was also an interaction between call type and time period (F_(4, 78.3)_ = 6.23, p = 0.0002). These findings strongly demonstrate that these non-vocal behaviors do not decrease in parallel with USV production during BBV playback.

The same group of non-vocal behaviors was compared using the same method across the group of males presented with white noise (Fig 8E–8H). Male time at the divider opening was the only non-vocal behavior to have a significant main effect of time period (F_(2,10.3)_ = 5.42, p = 0.0246), while digging (F_(2,13.2)_ = 0.41, p = 0.6744), grooming (F_(2,10.4)_ = 0.29, p = 0.7562), and female time at the divider opening (F_(2,11)_ = 1.65, p = 0.2352) did not significantly differ across time periods. The amount of time males spent at the divider opening did not change during the white noise playback but did increase to significantly greater than both baseline (t_17.9_ = -2.7, p = 0.0147), and during the white noise (t_8.99_ = -3.11, 0.0125) levels in the time period after the playback ended. By contrast, BBV playback resulted in an increase of digging during the playback and no change in investigation of the divider opening by the males.

**Fig 9:**
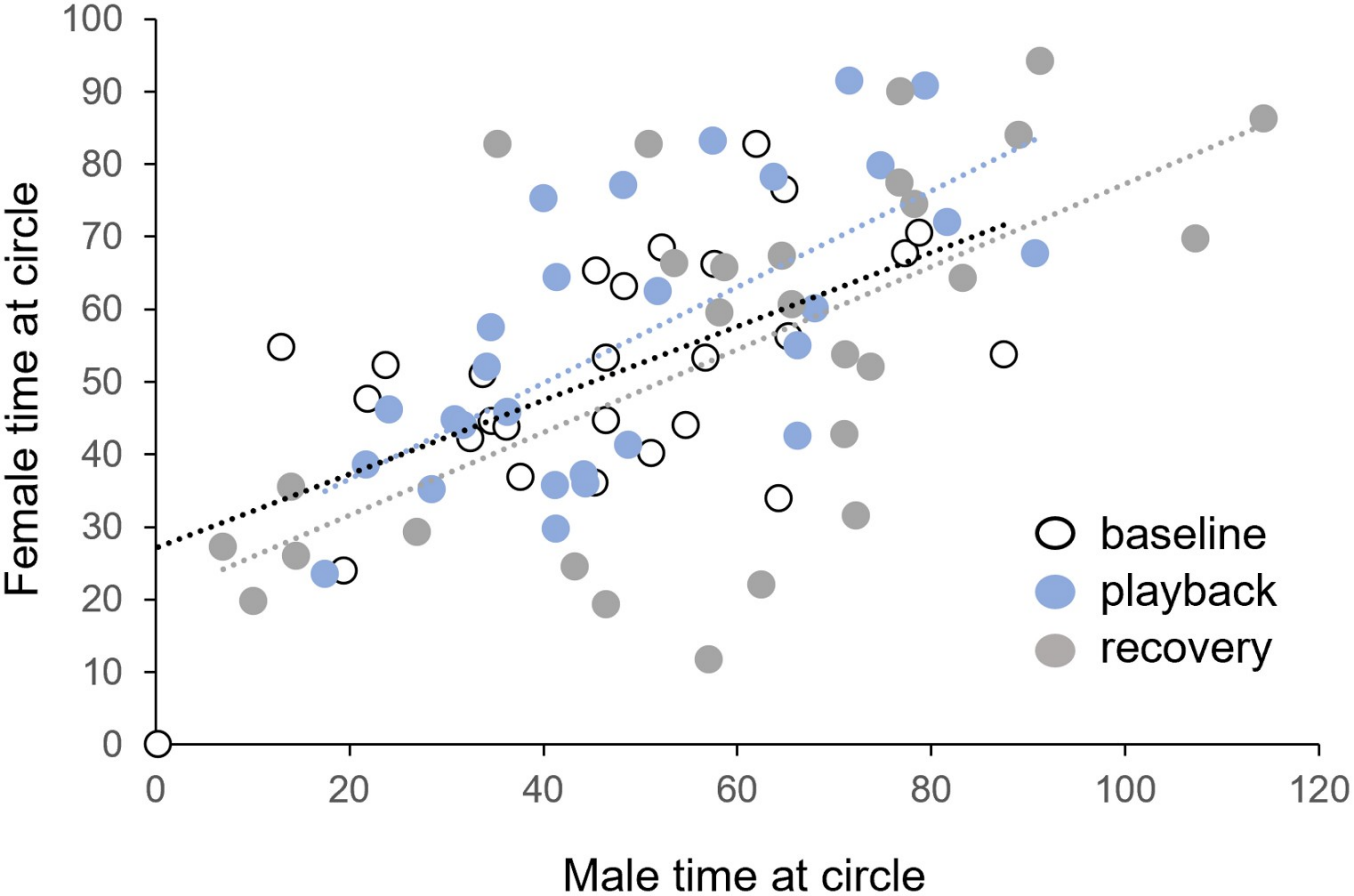
Correlation between male and female behavior. Although BBV playback does not alter male or female time spent at the circle, male and female time at the circle is correlated in the baseline period (white symbols), the playback period (blue circles), and the recovery period (gray circles). Dots represent individual trials including trials for all 3 exemplars (n = 28). Dashed lines indicate the linear regression for each period.

Although male but not female investigation changed during the recovery period, the amount of time that males and females spent investigating was related across individuals (Fig 9). This suggests that males and females were interacting at the divider opening, and co-investigation at the divider opening was often observed. Furthermore, the relationship between male and female investigation did not appear different in the baseline (open circle), playback (blue circle), or recovery (gray circle) time periods. To assess whether the relationship between male and female time at the divider opening was influenced by time period, we performed an analysis of covariance (ANCOVA) with female time at divider opening as the independent variable, male time at divider opening as the dependent variable, and time period (baseline, playback, and recovery) as a categorical variable. Although female time at divider opening predicted male time at divider opening (F_(1,77)_ = 46.54, p < 0.001), there was no interaction between female time at divider opening and time period (F_(2,77)_ = .024, p = 0.976). This result shows that male and female time at the divider opening were strongly correlated, but that this relationship did not differ across time periods.

## Discussion

The vocal signals of male vertebrates have been heavily studied as sexually selected behaviors, but females also produce vocalizations during opposite-sex interaction that serve a wide range of communicative functions. These vocalizations include ***advertisement calls*** that increase around times of high fertility or in response to ovarian hormones, ***copulation calls*** that may trigger competition among males, and ***vocal exchanges*** with males that may coordinate physiology and behavior or serve as territorial or mate defense [4, 27–32]. In addition to these, females may produce acoustically distinct calls associated with acute rejection, or ***dismissive or release calls*** [20, 33, 34]. Since dismissive calls may be coupled with defensive aggression [20, 35], it can be difficult to establish whether and how male receivers respond to these calls apart from their accompanying behaviors.

We have established a novel behavioral assay to assess male responses to a type of call (BBVs) made by female mice. Although both male and female mice produce USVs and BBVs in same-sex interactions, and BBVs in non-social distressing contexts, production of these signals is biased during opposite-sex interactions [35–38]. Males produce a majority of USVs in opposite-sex interactions, while females are thought to be the main source of BBVs [16, 19, 20, 35]. During opposite-sex interactions, females produce BBVs while performing physical rejection behaviors towards males, and BBVs produced in the early stages of an opposite-sex interaction correspond to a lack of male mounting at later stages of the interaction [17, 20]. The acoustic structure of BBVs may further encode information about individual identity, reproductive state, and receptivity [13, 17, 20]. These findings are consistent with BBVs acting as a dismissive signal by females, but do not distinguish a causal role for BBVs on male behavior from concomitant behaviors like kicking or lunging.

The novel split-cage paradigm we describe uses the presence of a female to evoke steady male USV production, but precludes females from making their own BBVs by preventing direct contact with males. Under these conditions, USVs decline, suggesting a causal role for BBVs in suppressing USVs in this paradigm. The suppression of USVs showed dynamics on several time scales. At the level of single BBVs, the probability of USVs declined rapidly and sharply, for less than a second. The speed of this response is comparable to the rapid exchange of vocal signals in rodents like male singing mice (*Scotinomys teguina* [39]), duetting birds like plain-tailed wrens (*Pheugopedius euophrys* [40]), or antiphonally calling amphibians like green tree frogs (*Hyla cinerea* [41]). Unlike these species there is no specific increase in the probability of call production immediately following the BBV-evoked suppression, so the responses of male mice are unlikely to represent an antiphonal vocal exchange. On a slightly longer time scale, the suppression of calls adapted over the rounds of playback. In species in which males show vocal suppression in response to female release calls like *Xenopus laevis*, adaptation on a similar time course is a hallmark of the responses to these female calls that distinguish them from the long-lasting suppression evoked by other species-specific calls [42, 43].

### The behavioral paradigm

Our behavioral assay does not replicate many of the aspects of a natural opposite sex interaction in mice. Chief among these is that it precludes contact between the males and females, an important behavior for collecting tactile and olfactory cues [44]. Our goal in the study was to create a relatively controlled environment based on our previous observations of vocal behavior in order to assess whether BBVs are capable of influencing male behavior when separated from co-occurring behaviors. In allowing us to treat BBVs as an independent experimental variable, this paradigm can also facilitate mechanistic studies of the auditory and related social neural systems in future.

However, the multimodal conditions of our paradigm, incorporating an awake female on one side of a barrier and the deposition of soiled female bedding on the male side of the barrier, did evoke high general levels of male vocal (USV) and nonvocal (investigative) engagement. The levels of USV production were comparable to those we have previously observed in direct interactions, although with a high level of inter-male variability in baseline calling, which is also observed in direct opposite sex interactions [17]. High baseline levels were important to be able to observe suppression of male responses as well as recovery from playback over a time scale of minutes.

The major result of our study, that BBV playback suppresses male USVs, is in accord with findings in two other behavioral paradigms. BBVs produced in the initial stages of a direct interaction inversely correspond to the production of USVs [17]. Additionally, when male mice are alone in an arena, playback of a mix of BBVs recorded from multiple females result in a lower probability of males producing USVs during playback [23]. These studies all suggest that under a range of social conditions, BBVs have the capacity to be suppressive to male vocal behavior.

### Role of the stimulus

Three separate exemplar BBVs were used in parallel experiments. These exemplars were not distinguishable from each other in the amount of USV suppression that they induced (Table 1). However, all exemplars were different from the control condition of silence, in which USVs were not suppressed. The lack of distinction among exemplars was the case even though exemplars had acoustic structures that differed in peak-to-peak and rms intensity, duration, and spectrotemporal structure (Fig 1). One similarity among exemplar playbacks is that they were all presented in the same temporal pattern, which replicated the timing of a one-minute bout recorded from a male-female interaction with a high level of BBV production accompanied by defensive aggression. This similarity among the effects of different calls suggests either that males respond to BBVs with different acoustic structure similarly, or else that the timing of the playback was the dominant factor in determining male responses. Similar characteristics are observed for the release calls (ticking calls) of female African clawed frogs, which vary in inter-call interval (ICI). Similar to BBVs in mice, ticking calls cause a suppression of male courtship calls. As long as the ICI is within the range of ticking calls, however, males suppress their courtship calls equally [42].

Similar to BBVs, the WN stimulus suppressed USVs, albeit more strongly. The similar responses of male to WN and BBVs can be viewed from several perspectives. The first of these assumes that the spectrotemporal characteristics of the BBVs are key to the suppression that we measured. From this perspective, WN may have replicated a behaviorally relevant component of natural BBVs, the inclusion of segments with high levels of deterministic chaos, which are the result of irregular vibration of the vocal cords [17, 45, 46]. In a range of mammalian species, vocalizations with non-linearities more consistently elicit anti-predator search behavior [47–49] and are less subject to habituation [50]. In house mice, the variation in BBV non-linear segments, specifically those segments made up of deterministic chaos, correspond to individual identity and estrous phase within individuals [17]. WN could thus potentially be perceived as a supernormal stimulus in this regard [51], exaggerating one of the most salient and information-rich portion of BBVs, the non-linear segments.

An alternative perspective is that both BBVs and WN are capable of suppressing USVs because they share the characteristics of being loud broadband signals. This interpretation is consistent with work from Blumstein et al. that found both American robins (*Turdus migratorius*) and warbling vireos (*Vireo gilvus*) were unable to discriminate between deterministic chaos and white noise [52]. An intriguing possibility is that the type of BBV that our assay replicates, consisting of loud broadband signals produced in close proximity to males, may be effective in part because they are startling. In some species, natural signals that either engage startle circuitry, or replicate naturally threatening stimuli, are effective sexual signals precisely because they exploit these effects [53, 54]. Our study did not address the neural circuitry underlying the responses of males to BBVs, but the possibility that our BBV playbacks could have startling qualities is consistent with the possibility that this may be an important aspect of BBVs produced during natural interactions.

At the same time, it is not true that playback of any signal type can suppress male vocalizations. The playback of different classes of vocalizations, female BBVs versus female USVs, to the same males, had different effects on vocal behavior, with female BBVs suppressing USVs and female USVs having no effect (Fig 4). This is similar to the result of a previous study from our lab in which BBVs and USVs were presented in factorial combination with female urine or a control [23]. BBV playback decreased the probability of male USV production, while USV playback in the presence of female urine increased the probability of male USV production, and the number of USVs produced, more than urine alone. Playback of vocalizations that are distinct from BBVs therefore produce different vocal responses by males. It is important to note that the USVs we played replicated the pattern of female USVs we observed in not only structure, but also timing and intensity. We therefore cannot distinguish whether any of these characteristics in particular were responsible for the difference in male responses to BBVs versus USVs.

### Role of the context

Both the immediate stimulus environment and prior social experience influenced the timecourse of male USV production. The assay described in this paper uses a live female as a stimulus partner for the focal male. Male mice produce USVs in response to female odor alone, and presenting female urine to male mice can increase the rate of USVs relative to baseline [23, 55–58]. When urine is the only stimulus, however, the increased rate of USVs can decline over time [23, 57]. Additionally, smaller USV responses occur in male mice presented with soiled female bedding or female urine as opposed to awake females [19]. Reflecting this pattern, males in the current study that were presented with either soiled female bedding or an anesthetized female showed a steady decline in USV production throughout the 15-minute recording period (Fig 2B). In contrast, males produced consistently high numbers of USVs throughout the entire recording when paired with an awake female. These findings suggest that stimuli combining olfactory cues with cues in additional uncharacterized modalities induce the most persistent male vocal behavior. Although males did not have direct access to females in the current study, they were able to contact females through a small hole in the acrylic barrier, and there was often nose-to-nose contact through this opening. We therefore cannot rule out the involvement of tactile cues in evoking persistent male calling.

Using awake females also introduced the variable of female behavior. It is possible that BBV playback may have influenced female behavior, but the female behavior that we measured, time at hole, was not different in the playback versus the baseline or recovery periods, while male BBV production was different in these periods. Another female behavior that we may have measured is production of USVs. Both males and females produce USVs during intersexual interactions, with females producing about a sixth of the total number of USVs [15, 59, 60]. There are minor structural differences between female and male USVs, but these may not always reliably identify the vocalizer’s sex [61, 62]. For this reason, we are unable to identify whether all USVs during playback recordings were produced by males. This is an important point, since some male behaviors correlate closely in time with female USVs, suggestive of responses to these female signals [60]. Since we could not definitively rule out the contribution of female USV production to the USV suppression by BBV playback, we presented an additional 12 males with either an anesthetized female or an anesthetized female combined with BBV playback. All males with relatively high calling rates in the first five minutes of the experiment showed sharp decreases in USV number in the first minute of the playback, and the decrease in USV number during playback was significantly different from baseline during BBV playback, but not when no playback was presented (Fig 7). This finding strongly suggests that even when females are unable to produce calls, males decrease USV production in response to playback.

Placing males in individual housing (social isolation) for a week prior to behavioral testing also altered the trajectory of USV production over the five rounds of BBV playback. Mice that have been isolated in the juvenile period alter their perception of calls in operant tests of call discrimination [63]. In the current study, socially housed males initially suppressed USVs to BBV playback but by Round 4 of the playback, their USV production had significantly increased compared to Round 1. Males housed individually showed a faster adaptation, showing increased USV numbers by Round 2 relative to Round 1. This is consistent with a general finding in mice that social isolation, whether in juvenile life or adulthood, alters vocal communication and perception. In particular, vocal behavior in mice that have been isolated in juvenile life is elevated, corresponding with increased non-vocal behaviors like anogenital investigation and same-sex mounting [64]. These findings are consistent with a lack of responsiveness to social signals in isolated mice. An alternative interpretation is based on the observation that, although isolated males did not have a significantly different baseline call rate than socially housed males in the current study, the baseline rate for isolated males was quantitatively elevated. The different trajectory of BBVs for isolated males during playback rounds could therefore have been influenced by a *greater* initial response by isolated males relative to baseline. Along with studies showing that mice vocalize differently depending on their immediately preceding social context [59], these findings emphasize that prior social experience is an important lens through which mice perceive current social interactions.

### Role of BBVs in male-female interaction

Studies of vocal communication in opposite-sex contexts are often focused on male signals, but there is a growing body of evidence that courtship is a dynamic interaction between males and females that mutually vocally signal [65, 66]. Female mouse BBVs are made during courtship and have the potential to communicate multiple types of information [17]. Our results support the hypothesis that BBVs modify male behavior during male-female interactions. Specifically, during BBV playback, males suppress the production of pro-social USVs.

BBVs, in conjunction with other behaviors such as female kicking and lunging, could potentially play a role in pacing courtship interactions. In rats, females that are allowed to pace mating by having access to a section of the cage the male cannot reach are more likely to become pregnant as a result of mating and will display a conditioned place preference to the mating cage [12, 67]. There is some evidence that house mice use a similar pacing strategy by either physically escaping males or by defensive aggression, but it has not been as widely described as paced mating in rats [13, 14]. BBVs could modify the timecourse of opposite sex interaction in mice by reinforcing nonvocal rejection behaviors by females, or by acting as a signal that reduces male vocalizing. Supporting this view, when female mice can escape males up the side of a mesh cage, BBV production escalates in the 10 seconds immediately preceding escape, and no mounting by males occurs [17].

Despite the evidence for vocal suppression by BBVs in the current study, there is strong evidence that BBVs do not play a static behavioral role. Across multiple studies and even within single studies, BBVs have been associated with non-vocal rejection behaviors by females but also with male mounting, with the different behavioral combinations associated with early/appetitive versus later/consummatory phases of interactions [17, 20, 33] Across playback studies that have included BBVs or equivalent calls, the responses of males to playback have also varied with the signal context. For male mice alone in an arena, the playback of BBVs reduces the probability of males producing USVs in response to the odor of female urine, but also causes the USVs triggered by urine to persist for a longer period of time than the presentation of urine alone [23]. Grimsley et al. (2013) likewise demonstrated that male mice do not show aversion to BBVs (called low frequency harmonics, or LFHs, in this study) when they are paired with female urine, but do show aversion when LFHs are paired with cat hair. The playback of LFHs can also cause an increase rather than a decrease in USVs produced by either males or females alone in an arena [68]. Finally, in addition to being produced in conjunction with defensive aggression early in opposite-sex interactions, BBVs are also produced in a later time period as females are mounted by males [17, 19]. It is therefore likely that although our current behavioral assay captured a socially suppressive aspect of BBVs, this is just one aspect of these interesting signals.

## Supporting information

S1 Fig Structure of the USV stimuli used in the study. **A)** Oscillogram indicating the locations of ten clusters of six female USVs each played over a 5-minute period. **B)** Spectrograms of representative USVs used in the USV clusters.
(TIF)

## References

[1] Amy M, Salvin P, Naguib M, Leboucher G. Female signalling to male song in the domestic canary, *Serinus canaria*. R Soc open sci. 2015;2: 140196. doi: 10.1098/rsos.140196 26064577 PMC4448791

[2] Briceño RD, Eberhard WG. Copulatory Dialogues Between Male and Female Tsetse Flies (Diptera: Muscidae: Glossina pallidipes). J Insect Behav. 2017;30: 394–408. doi: 10.1007/s10905-017-9625-1

[3] Gregory Byrne P. Strategic Male Calling Behavior in an Australian Terrestrial Toadlet (Pseudophryne Bibronii). Copeia. 2008;2008: 57–63. doi: 10.1643/CE-05-294

[4] Langmore NE. Functions of duet and solo songs of female birds. Trends in Ecology & Evolution. 1998;13: 136–140. doi: 10.1016/s0169-5347(97)01241-x 21238233

[5] Løvlie H, Zidar J, Berneheim C. A cry for help: female distress calling during copulation is context dependent. Animal Behaviour. 2014;92: 151–157. doi: 10.1016/j.anbehav.2014.04.002

[6] Peretti A, Eberhard WG, Briceño RD. Copulatory dialogue: female spiders sing during copulation to influence male genitalic movements. Animal Behaviour. 2006;72: 413–421. doi: 10.1016/j.anbehav.2006.01.014

[7] Rodríguez RL, Haen C, Cocroft RB, Fowler-Finn KD. Males adjust signaling effort based on female mate-preference cues. Behavioral Ecology. 2012;23: 1218–1225. doi: 10.1093/beheco/ars105

[8] Rodríguez RL. Mating Is a Give-and-Take of Influence and Communication Between the Sexes. In: Peretti AV, Aisenberg A, editors. Cryptic Female Choice in Arthropods. Cham: Springer International Publishing; 2015. pp. 479–496. doi: 10.1007/978-3-319-17894-3_18

[9] Patricelli GL, Uy JAC, Walsh G, Borgia G. Male displays adjusted to female’s response. Nature. 2002;415: 279–280. doi: 10.1038/415279a 11796996

[10] Patricelli GL, Coleman SW, Borgia G. Male satin bowerbirds, Ptilonorhynchus violaceus, adjust their display intensity in response to female startling: an experiment with robotic females. Animal Behaviour. 2006;71: 49–59. doi: 10.1016/j.anbehav.2005.03.029

[11] Noble RG. Sex responses of the female hamster: Effects on male performance. Physiology & Behavior. 1980;24: 237–242. doi: 10.1016/0031-9384(80)90080-3 7375538

[12] Frye CA, Erskine MS. Influence of time of mating and paced copulation on induction of pseudopregnancy in cyclic female rats. Reproduction. 1990;90: 375–385. doi: 10.1530/jrf.0.0900375 2250236

[13] Johansen JA, Clemens LG, Nunez AA. Characterization of copulatory behavior in female mice: Evidence for paced mating. Physiology & Behavior. 2008;95: 425–429. doi: 10.1016/j.physbeh.2008.07.004 18662708 PMC2574982

[14] Garey J, Kow L-M, Huynh W, Ogawa S, Pfaff DW. Temporal and Spatial Quantitation of Nesting and Mating Behaviors among Mice Housed in a Semi-Natural Environment. Hormones and Behavior. 2002;42: 294–306. doi: 10.1006/hbeh.2002.1823 12460589

[15] Neunuebel JP, Taylor AL, Arthur BJ, Egnor SR. Female mice ultrasonically interact with males during courtship displays. eLife. 2015;4: e06203. doi: 10.7554/eLife.06203 26020291 PMC4447045

[16] Warren MR, Spurrier MS, Roth ED, Neunuebel JP. Sex differences in vocal communication of freely interacting adult mice depend upon behavioral context. Cooper BG, editor. PLoS ONE. 2018;13: e0204527. doi: 10.1371/journal.pone.0204527 30240434 PMC6150532

[17] Finton CJ, Keesom SM, Hood KE, Hurley LM. What’s in a squeak? Female vocal signals predict the sexual behaviour of male house mice during courtship. Animal Behaviour. 2017;126: 163–175. doi: 10.1016/j.anbehav.2017.01.021

[18] Grimsley JMS, Hazlett EG, Wenstrup JJ. Coding the Meaning of Sounds: Contextual Modulation of Auditory Responses in the Basolateral Amygdala. Journal of Neuroscience. 2013;33: 17538–17548. doi: 10.1523/JNEUROSCI.2205-13.2013 24174686 PMC3812514

[19] Wang H, Liang S, Burgdorf J, Wess J, Yeomans J. Ultrasonic Vocalizations Induced by Sex and Amphetamine in M2, M4, M5 Muscarinic and D2 Dopamine Receptor Knockout Mice. Baune B, editor. PLoS ONE. 2008;3: e1893. doi: 10.1371/journal.pone.0001893 18382674 PMC2268741

[20] Sugimoto H, Okabe S, Kato M, Koshida N, Shiroishi T, Mogi K, et al. A Role for Strain Differences in Waveforms of Ultrasonic Vocalizations during Male–Female Interaction. de Polavieja GG, editor. PLoS ONE. 2011;6: e22093. doi: 10.1371/journal.pone.0022093 21818297 PMC3144874

[21] Lindzey G, Manosevitz M, Winston H. Social dominance in the mouse. Psychon Sci. 1966;5: 451–452. doi: 10.3758/BF03331044

[22] Wang F, Kessels HW, Hu H. The mouse that roared: neural mechanisms of social hierarchy. Trends in Neurosciences. 2014;37: 674–682. doi: 10.1016/j.tins.2014.07.005 25160682

[23] Ronald KL, Zhang X, Morrison MV, Miller R, Hurley LM. Male mice adjust courtship behavior in response to female multimodal signals. Matsunami H, editor. PLoS ONE.2020;15: e0229302. doi: 10.1371/journal.pone.0229302 32241020 PMC7117945

[24] Hanson JL, Hurley LM. Female Presence and Estrous State Influence Mouse Ultrasonic Courtship Vocalizations. Bolhuis JJ, editor. PLoS ONE. 2012;7: e40782. doi: 10.1371/journal.pone.0040782 22815817 PMC3399843

[25] Benjamini Y, Hochberg Y. Controlling the False Discovery Rate: A Practical and Powerful Approach to Multiple Testing. Journal of the Royal Statistical Society: Series B (Methodological). 1995;57: 289–300. doi: 10.1111/j.2517-6161.1995.tb02031.x

[26] Freiler MK, Proffitt MR, Smith GT. Electrocommunication signals and aggressive behavior vary among male morphs in an apteronotid fish, Compsaraia samueli. J Exp Biol. 2022;225: jeb243452. 10.1242/jeb.243452 PMC9250798 35603444

[27] Matochik JA, White NR, Barfield RJ. Variations in scent marking and ultrasonic vocalizations by Long-Evans rats across the estrous cycle. Physiology & Behavior. 1992;51: 783–786. doi: 10.1016/0031-9384(92)90116-j 1594676

[28] Semple S, McComb K, Alberts S, Altmann J. Information content of female copulation calls in yellow baboons. Am J Primatol. 2002;56: 43–56. doi: 10.1002/ajp.1062 11793412

[29] Leong KM, Ortolani A, Graham LH, Savage A. The use of low-frequency vocalizations in African elephant(Loxodonta africana) reproductive strategies. Hormones and Behavior. 2003;43: 433–443. doi: 10.1016/s0018-506x(03)00025-4 12788289

[30] Ellis JMS, Langen TA, Berg EC. Signalling for food and sex? Begging by reproductive female white-throated magpie-jays. Animal Behaviour. 2009;78: 615–623. doi: 10.1016/j.anbehav.2009.05.024 23293376 PMC3534969

[31] Charlton BD, Keating JL, Rengui L, Huang Y, Swaisgood RR. Female giant panda (*Ailuropoda melanoleuca*) chirps advertise the caller’s fertile phase. Proc R Soc B. 2010;277: 1101–1106. doi: 10.1098/rspb.2009.1431 19955154 PMC2842754

[32] Clay Z, Pika S, Gruber T, Zuberbühler K. Female bonobos use copulation calls as social signals. Biol Lett. 2011;7: 513–516. doi: 10.1098/rsbl.2010.1227 21325305 PMC3130230

[33] Kelley DB. Female sex behaviors in the South African clawed frog, Xenopus laevis: Gonadotropin-releasing, gonadotropic, and steroid hormones. Hormones and Behavior. 1982;16: 158–174. doi: 10.1016/0018-506x(82)90016-2 6749641

[34] Charlton BD. The Acoustic Structure and Information Content of Female Koala Vocal Signals. Slocombe KE, editor. PLoS ONE. 2015;10: e0138670. doi: 10.1371/journal.pone.0138670 26465340 PMC4605621

[35] White NR, Prasad M, Barfield RJ, Nyby JG. 40- and 70-kHz Vocalizations of Mice (Mus musculus) during Copulation. Physiology & Behavior. 1998;63: 467–473. doi: 10.1016/s0031-9384(97)00484-8 9523885

[36] Shintomi K. Effects of psychotropic drugs on methamphetamine-induced behavioral excitation in grouped mice. European Journal of Pharmacology. 1975;31: 195–206. doi: 10.1016/0014-2999(75)90040-0 238852

[37] Hammerschmidt K, Reisinger E, Westekemper K, Ehrenreich L, Strenzke N, Fischer J. Mice do not require auditory input for the normal development of their ultrasonic vocalizations. BMC Neurosci. 2012;13: 40. doi: 10.1186/1471-2202-13-40 22533376 PMC3350408

[38] Hoier S, Pfeifle C, von Merten S, Linnenbrink M. Communication at the Garden Fence–Context Dependent Vocalization in Female House Mice. Koide T, editor. PLoS ONE. 2016;11: e0152255. doi: 10.1371/journal.pone.0152255 27022749 PMC4811528

[39] Okobi DE, Banerjee A, Matheson AMM, Phelps SM, Long MA. Motor cortical control of vocal interaction in neotropical singing mice. Science. 2019;363: 983–988 doi: 10.1126/science.aau9480 30819963

[40] Coleman M, Fortune E. Duet singing in plain-tailed wrens. Curr Biol. 2018;28: R643–R645. doi: 10.1016/j.cub.2018.02.066 29870698

[41] Jones DL, Jones RL, Ratnam R. Calling dynamics and call synchronization in a local group of unison bout callers. J Comp Physiol A Neuroethol Sens Neural Behav Physiol. 2014;200: 93–107. doi: 10.1007/s00359-013-0867-x 24249152

[42] Elliott TM, Kelley DB. Male discrimination of receptive and unreceptive female calls by temporal features. Journal of Experimental Biology. 2007;210: 2836–2842. doi: 10.1242/jeb.003988 17690231 PMC3493248

[43] Hall IC, Ballagh IH, Kelley DB. The Xenopus Amygdala Mediates Socially Appropriate Vocal Communication Signals. Journal of Neuroscience. 2013;33: 14534–14548. doi: 10.1523/JNEUROSCI.1190-13.2013 24005304 PMC3761055

[44] Ramm SA, Cheetham SA, Hurst JL. Encoding choosiness: female attraction requires prior physical contact with individual male scents in mice. Proceedings of the Royal Society B. 2008;275: 1727–1735. doi: 10.1098/rspb.2008.0302 18448415 PMC2587794

[45] Fitch WT, Neubauer J, Herzel H. Calls out of chaos: the adaptive significance of nonlinear phenomena in mammalian vocal production. Animal Behaviour. 2002;63: 407–418. doi: 10.1006/anbe.2001.1912

[46] Lupanova AS, Egorova MA. Vocalization of sex partners in the house mouse (Mus Musculus). J Evol Biochem Phys. 2015;51: 324–331. doi: 10.1134/S0022093015040080 26547953

[47] Ruiz-Monachesi MR, Labra A. Complex distress calls sound frightening: the case of the weeping lizard. Animal Behaviour. 2020;165: 71–77. doi: 10.1016/j.anbehav.2020.05.004

[48] Slaughter EI, Berlin ER, Bower JT, Blumstein DT. A Test of the Nonlinearity Hypothesis in Great-tailed Grackles (*Quiscalus mexicanus*). Foster S, editor. Ethology. 2013;119: 309–315. doi: 10.1111/eth.12066

[49] Yan KM, Pinto SP, Vartany C, Blumstein DT. Shift down, look up: A test of the non-linearity and fear hypothesis in a non-vocal skink. Schneider J, editor. Ethology. 2019;125: 153–158. doi: 10.1111/eth.12839

[50] Karp D, Manser MB, Wiley EM, Townsend SW. Nonlinearities in Meerkat Alarm Calls Prevent Receivers from Habituating. Fusani L, editor. Ethology. 2014;120: 189–196. doi: 10.1111/eth.12195

[51] Tinbergen N, Perdeck AC. On the Stimulus Situation Releasing the Begging Response in the Newly Hatched Herring Gull Chick (Larus Argentatus Argentatus Pont.). Behav. 1951;3: 1–39. doi: 10.1163/156853951X00197

[52] Blumstein DT, Whitaker J, Kennen J, Bryant GA. Do birds differentiate between white noise and deterministic chaos? Koenig W, editor. Ethology. 2017;123: 966–973. doi: 10.1111/eth.12702

[53] Ter Hofstede HM, Schöneich S, Robillard T, Hedwig B. Evolution of a Communication System by Sensory Exploitation of Startle Behavior. Curr Biol. 2015;25:3245–52. doi: 10.1016/j.cub.2015.10.064 26687622

[54] Nakano R, Takanashi T, Skals N, Surlykke A, Ishikawa Y. To females of a noctuid moth, male courtship songs are nothing more than bat echolocation calls. Biol Lett. 2010;6:582–584. doi: 10.1098/rsbl.2010.0058 20219743 PMC2936132

[55] Whitney G, Coble JR, Stockton MD, Tilson EF. Ultrasonic emissions: Do they facilitate courtship of mice? Journal of Comparative and Physiological Psychology. 1973;84: 445–452. doi: 10.1037/h0034899 4745813

[56] Nyby J, Wysocki CJ, Whitney G, Dizinno G. Pheromonal regulation of male mouse ultrasonic courtship (Mus musculus). Animal Behaviour. 1977;25: 333–341. doi: 10.1016/0003-3472(77)90009-4 889149

[57] Dizinno G, Whitney G, Nyby J. Ultrasonic vocalizations by male mice (Mus musculus) to female sex pheromone: Experiential determinants. Behavioral Biology. 1978;22: 104–113. doi: 10.1016/S0091-6773(78)92094-1

[58] Nyby J, Zakeski D. Elicitation of male mouse ultrasounds: Bladder urine and aged urine from females. Physiology & Behavior. 1980;24: 737–740. doi: 10.1016/0031-9384(80)90405-9 7394016

[59] Burke K, Screven LA, Dent ML. CBA/CaJ mouse ultrasonic vocalizations depend on prior social experience. Rosenfeld CS, editor. PLoS ONE. 2018;13: e0197774. doi: 10.1371/journal.pone.0197774 29874248 PMC5991354

[60] Warren MR, Clein RS, Spurrier MS, Roth ED, Neunuebel JP. Ultrashort-range, high-frequency communication by female mice shapes social interactions. Sci Rep. 2020;10: 2637. doi: 10.1038/s41598-020-59418-0 32060312 PMC7021676

[61] Hammerschmidt K, Radyushkin K, Ehrenreich H, Fischer J. The Structure and Usage of Female and Male Mouse Ultrasonic Vocalizations Reveal only Minor Differences. Bolhuis JJ, editor. PLoS ONE. 2012;7: e41133. doi: 10.1371/journal.pone.0041133 22815941 PMC3398926

[62] Ivanenko A, Watkins P, van Gerven MAJ, Hammerschmidt K, Englitz B. Classifying sex and strain from mouse ultrasonic vocalizations using deep learning. Theunissen FE, editor. PLoS Comput Biol. 2020;16: e1007918. doi: 10.1371/journal.pcbi.1007918 32569292 PMC7347231

[63] Screven LA, Dent ML. Perception of Ultrasonic Vocalizations by Socially Housed and Isolated Mice. eNeuro. 2019;6: ENEURO.0049-19.2019. doi: 10.1523/ENEURO.0049-19.2019 31570420 PMC6794080

[64] Keesom SM, Finton CJ, Sell GL, Hurley LM. Early-Life Social Isolation Influences Mouse Ultrasonic Vocalizations during Male-Male Social Encounters. Coleman MJ, editor. PLoS ONE. 2017;12: e0169705. doi: 10.1371/journal.pone.0169705 28056078 PMC5215938

[65] Guillermo-Ferreira R, Bispo PC. Male and female interactions during courtship of the Neotropical damselfly Mnesarete pudica (Odonata: Calopterygidae). acta ethol. 2012;15: 173–178. doi: 10.1007/s10211-012-0122-4

[66] Coleman SW, Patricelli GL, Borgia G. Variable female preferences drive complex male displays. Nature. 2004;428: 742–745. doi: 10.1038/nature02419 15085130

[67] Coopersmith C, Erskine MS. Influence of paced mating and number of intromissions on fertility in the laboratory rat. Reproduction. 1994;102: 451–458. doi: 10.1530/jrf.0.1020451 7861400

[68] Niemczura AC, Grimsley JM, Kim C, Alkhawaga A, Poth A, Carvalho A, et al. Physiological and Behavioral Responses to Vocalization Playback in Mice. Front Behav Neurosci. 2020;14: 155. doi: 10.3389/fnbeh.2020.00155 33033474 PMC7490332

