# Supplementary figures and images for: Playback of broadband vocalizations of female mice suppresses male ultrasonic calls
Source: PLoS One. 2023 Jan 5;18(1):e0273742. doi: 10.1371/journal.pone.0273742 (PMC9815654; doi:10.1371/journal.pone.0273742)

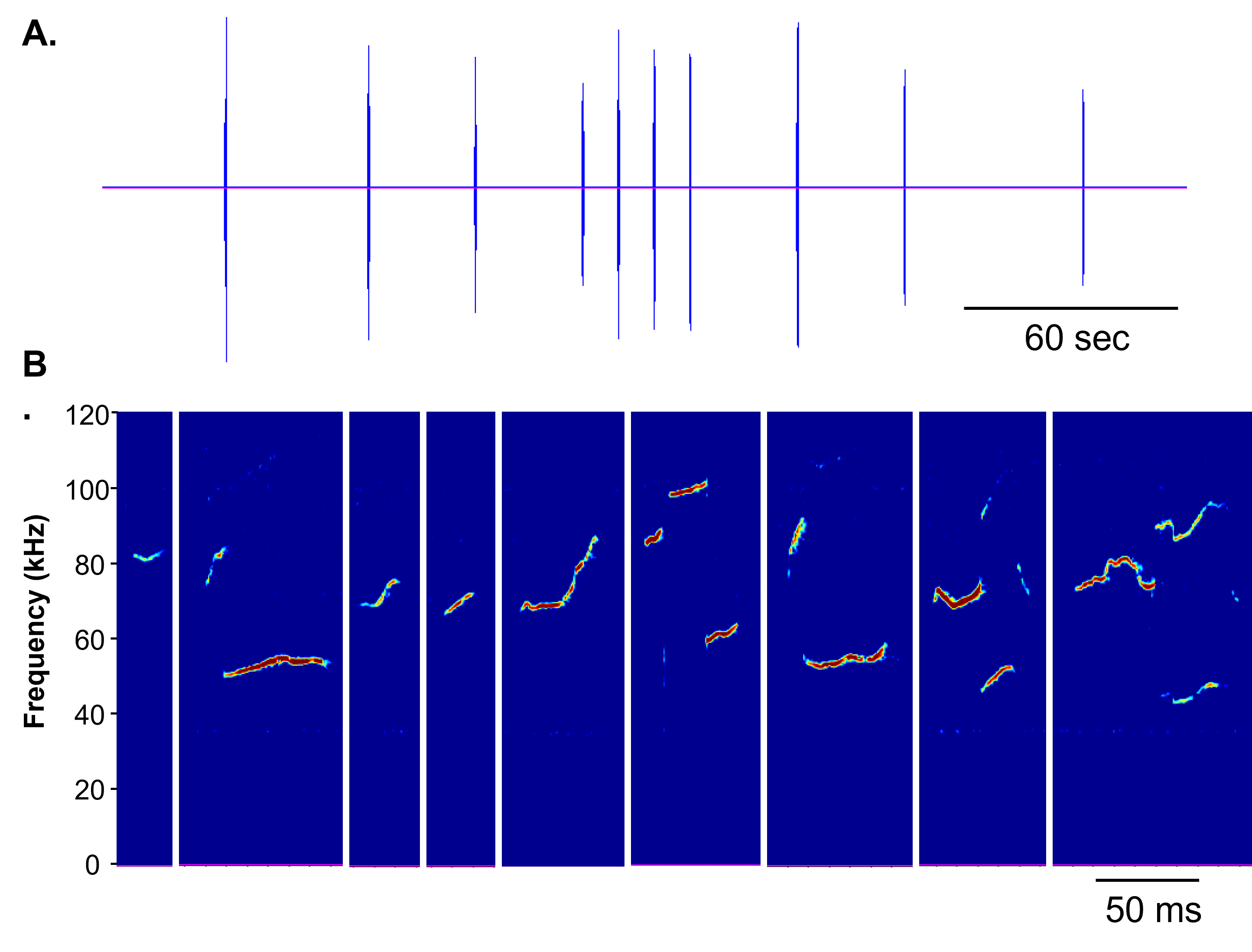

Supplement: S1 Fig — A) Oscillogram indicating the locations of ten clusters of six female USVs each played over a 5-minute period. B) Spectrograms of representative USVs used in the USV clusters. (TIF) [file pone.0273742.s001.tif]
